# The retrograde IFT dynein is required for normal function of diverse mechanosensory cilia in *Drosophila*

**DOI:** 10.3389/fnmol.2023.1263411

**Published:** 2023-09-22

**Authors:** Yashoda Sharma, Julie S. Jacobs, Elena Sivan-Loukianova, Eugene Lee, Maurice J. Kernan, Daniel F. Eberl

**Affiliations:** ^1^Department of Biology, The University of Iowa, Iowa City, IA, United States; ^2^Department of Neurobiology and Behavior, State University of New York, Stony Brook, NY, United States

**Keywords:** cilia, intraflagellar transport, cytoplasmic dynein 1b, hearing, *Drosophila*, aging, degeneration

## Abstract

**Introduction:**

Cilia biogenesis relies on intraflagellar transport (IFT), a conserved transport mechanism which functions bi-directionally to bring protein complexes to the growing ciliary tip and recycle signaling and transport proteins between the cilium and cell body. In *Drosophila*, anterograde IFT is critical for assembly of sensory cilia in the neurons of both chordotonal (ch) organs, which have relatively long ciliary axonemes, and external sensory (es) organs, which have short axonemal segments with microtubules in distal sensory segments forming non-axonemal bundles. We previously isolated the *beethoven* (*btv*) mutant in a mutagenesis screen for auditory mutants. Although many *btv* mutant flies are deaf, some retain a small residual auditory function as determined both by behavior and by auditory electrophysiology.

**Results:**

Here we molecularly characterize the *btv* gene and demonstrate that it encodes the IFT-associated dynein-2 heavy chain Dync2h1. We also describe morphological changes in Johnston’s organ as flies age to 30 days, and we find that morphological and electrophysiological phenotypes in this ch organ of *btv* mutants become more severe with age. We show that NompB protein, encoding the conserved IFT88 protein, an IFT complex B component, fails to be cleared from chordotonal cilia in *btv* mutants, instead accumulating in the distorted cilia. In macrochaete bristles, a class of es organ, *btv* mutants show a 50% reduction in mechanoreceptor potentials.

**Discussion:**

Thus, the *btv*-encoded Dync2h1 functions as the retrograde IFT motor in the assembly of long ciliary axonemes in ch organs and is also important for normal function of the short ciliary axonemes in es organs.

## Introduction

The many cellular functions of cilia include motility of unicellular organisms and spermatozoa, the streaming of extracellular fluids, and signal transduction in development, homeostasis, and sensory perception. Ciliary dysfunction is associated with a correspondingly diverse set of symptoms in human disorders, such as primary ciliary dyskinesia, polycystic kidney disease, Bardet-Biedl syndrome, Joubert syndrome, and collectively termed ciliopathies (Sharma et al., 2008; Hildebrandt et al., 2011).

All cilia and membrane-enclosed flagella require intraflagellar transport (IFT) for their assembly and maintenance [reviewed by Rosenbaum and Witman (2002); Ishikawa and Marshall (2017); Pigino (2021)]. IFT is a bidirectional mechanism that brings axoneme components to the growing tip of the cilium or flagellum and recycles signaling and transport proteins between the cilium and cell body. Movement in each direction is powered by a different processive motor protein, traveling on the polarized microtubules of the axoneme. Anterograde transport, towards the tip, is driven by plus end-directed kinesin II (Cole et al., 1998); retrograde transport is driven by a specialized cytoplasmic-type dynein complex, dynein-2 (Vuolo et al., 2020). IFT was first observed in *Chlamydomonas* as the uninterrupted movement of trains of particles along the flagellum, in the space between the axoneme and flagellar membrane (Kozminski et al., 1993). The trains include two multiprotein complexes, A and B (Cole et al., 1998), respectively composed of 6 and 16 polypeptides, most of which are conserved across all ciliated eukaryotes. The IFT-A and IFT-B complexes serve as scaffolds to organize and regulate the motors, ensuring uninterrupted movement in each direction. They are also sites of interaction with axonemal cargoes and with adaptors, such as Tulp and the BBSome complex, for ciliary membrane proteins (Jordan and Pigino, 2021; Lechtreck, 2022).

Recent studies employing cryo-electron microscopy, protein cross-linking and computational structure prediction have produced models of the A and B complexes (Hesketh et al., 2022; Meleppattu et al., 2022; Petriman et al., 2022), and of their arrangement in anterograde trains (Lacey et al., 2023). The trains have a double-decked profile, with repeated IFT-B complexes aligned next to the axoneme, overlaid by IFT-A complexes next to the ciliary membrane. Dynein-2 is carried in anterograde trains as cargo attached to the B complex, held away from the axonemal microtubules with its motor subunits in an autoinactivated configuration (Jordan et al., 2018). The retrograde train structure has not been worked out to this resolution but is clearly different in its architecture (Stepanek and Pigino, 2016), indicating that the IFT trains undergo major reconfiguration at the ciliary tip, releasing cargo, inactivating kinesin and bringing dynein-2 into play (Chien et al., 2017).

Mutations affecting motor or IFT complex proteins show ciliary phenotypes consistent with specific defects in anterograde or retrograde transport. Mutants lacking kinesin II activity or required IFT-B proteins show no anterograde transport and typically have very truncated or no cilia flagella (Cole et al., 1998; Marszalek et al., 2000; Parker and Quarmby, 2003; Sarpal et al., 2003). But in mutants with defects in dynein-2 or IFT-A proteins, anterograde transport can proceed until halted by the lack of recycled components, producing cilia that are longer but distended or distorted, in which IFT components accumulate (Criswell et al., 1996; Criswell and Asai, 1998; Pazour et al., 1999; Signor et al., 1999; Wicks et al., 2000; Mikami et al., 2002). Mutations in human *DYNC2H1*, the heavy chain subunit of dynein-2, are associated with Asphyxiating Thoracic Dystrophy (ATD) and Short-rib Polydactyly Syndrome (SRP) Type III (Dagoneau et al., 2009; Merrill et al., 2009), conditions that are consistent with ciliary dysfunction.

In *Drosophila*, ciliated cells include spermatozoa and Type I sensory neurons. Sensory neurons in chordotonal (ch) organs (also called scolopidia) exhibit long cilia with an electron-dense ciliary dilation about three fourths of the length from basal body to ciliary tip. Conversely, bristle, campaniform and olfactory organ neurons have very short ciliary segments at the basal bodies, with loosely organized distal non-axonemal microtubule bundles. Surprisingly, although IFT is required to build sensory cilia, sperm tail assembly and maintenance in *Drosophila* appear to be independent of IFT. This conclusion is based on studies with the kinesin associated protein (DmKAP) and the motor subunit Klp64D (Sarpal et al., 2003) as well as with NompB (Han et al., 2003), the *Drosophila* homolog of IFT88/Polaris/OSM-5 (Pazour et al., 2002) and RempA, the homolog of the complex A protein IFT140 (Lee et al., 2008). In *Drosophila*, many genes required for ciliary assembly are activated by the Rfx transcription factor (Durand et al., 2000; Vandaele et al., 2001; Laurençon et al., 2007) and the forkhead-domain transcription factor Fd3F (Newton et al., 2012), but while Rfx is expressed in elongating stage spermatid nuclei (Vandaele et al., 2001) there have been no reports that either Rfx or Fd3F are required for male fertility.

Chordotonal organs participate in several sensory modalities. In *Drosophila* larvae, ch organs provide touch-sensitivity and proprioceptive feedback during locomotion (Kernan et al., 1994; Caldwell et al., 2003). They have also been shown to be receptive to auditory stimuli (Zhang et al., 2013; Li et al., 2021). In addition, some ch neurons are among the thermosensory neurons in the larva (Liu et al., 2003; Kwon et al., 2010). In the adult, ch organs located in the limbs and abdomen include both proprioceptive and vibrosensory neurons (Fabre et al., 2012; Mamiya et al., 2018). Wing ch organs provide feedback not only during flight, but also during courtship song production (Tauber and Eberl, 2001). Johnston’s organ (JO), the antennal ch organ, functions as the auditory organ (Eberl et al., 2000). It is also responsible for gravity and wind sensing (Armstrong et al., 2006; Kamikouchi et al., 2009; Sun et al., 2009; Yorozu et al., 2009). Chordotonal organs in the adult also contribute to temperature entrainment of adult circadian rhythms, though JO is not required for this function (Sehadova et al., 2009).

The phenotypes we previously described for the *beethoven* (*btv*) mutation (Eberl et al., 1997, 2000; Tauber and Eberl, 2001; Caldwell et al., 2003), together with mapping data, suggested that Dync2h1, the retrograde motor for *Drosophila* IFT, was a good candidate gene. Here we demonstrate that *btv* is the predicted gene *CG15148*, which encodes the Dync2h1 homolog. We show that *btv* is required for ciliary assembly in ch organs, and that morphological and electrophysiological phenotypes of *btv* mutants become more severe with age. To support the notion that the Dync2h1 encoded by *btv* actually participates in retrograde IFT, we show that NompB, an IFT complex B protein, fails to be cleared from the chordotonal cilium in *btv* mutants. Finally, we show that the *btv* mutation also reduces mechanoreceptor potentials (MRPs) of bristle organs by about 50% without affecting trans-epithelial potentials (TEPs), suggesting that retrograde IFT plays a role in external sensory (es) organs despite the shortness of these axonemal segments (Table 1).

**Table 1:** Genetic variants used in this study.

*btv* genotype	Synonyms	Molecular defect	DZA score	Hearing	Remarks	References
btv^+^	40AG13	None		Normal	Control for btv^1^	Eberl et al. (1997, 2000)
btv^1^	btv^5P1^	401 bp deletion/6 bp insertion, intron 12–13 into exon 13	4.79	Severe	EMS-induced	Eberl et al. (1997, 2000); this work
btv^2^	btv^k07109b^	Frameshift caused by single nucleotide deletion of A in exon 22, coordinate 17,966,613		Severe	P-induced; not associated with P-inserts	Spradling et al. (1999), Mancebo et al. (2001), Bellen et al. (2004) and Comeron et al. (2016); this work
Df(2 L)TW119/Df(2 L)TW201		Homozygous deficient from *CadN2* to *rdo*	5.0; 5.4	Severe	Viable, and show rdo phenotype; male sterile due to other loci	Eberl et al. (2000)
btv^3^	BG01771	P-insert, intron 13–14	1.5; 1.0; 1.89	Normal	P{GT1} insertion (dual-tag)	Bellen et al. (2004); this work
btv^4^	btv^Lf234^	3,196 bp deletion of exons 12–13; 1 bp insertion (G)	5.0	Severe	P-induced; 2nd-site lethal; tested over btv^1^	This work
btv^5^	f06884	PBac insert, exon 23		Severe	PBac{WH} insertion	Thibault et al. (2004)
btv^6^	f06878	PBac insert, intron 13–14		Normal	PBac{WH} insertion, 6 bp left of the BG01771 insertion site	Thibault et al. (2004)
del#1		Deletion of DNA between f06884 and f06319 insertion sites		Severe	deletes 3′ end of Dync2h1 leftward	This work
del#2		Deletion of DNA between f06878 and f06603 insertion sites		Severe	deletes 5′ end of Dync2h1 rightward	This work

## Materials and methods

### Fly strains and crosses

*Drosophila melanogaster* mutants of *btv* used in this study are listed in Table 1. For P-induced male recombination, the transposase source was supplied by *w^+^*; *Sp btv^1^ pr rl cn*/*CyO*; *Dr P {Δ2-3}*/*TM6, Ubx*. The *Sternopleural* (*Sp*) and *purple* (*pr*) genes are used as flanking markers to map *btv*. These flies were crossed to *w/Y; P {GT1}BG01771* males. Dysgenic male offspring were crossed to *w^+^*; *Df* (*2 L*)*TW12 Tft pr*/*CyO*, *pr* females to recover *Sp pr^+^* or *Sp^+^ pr* recombinants. Recombinants were subsequently tested for btv genotype by crossing to *w*; *btv^1^* 40AG13/*CyO*. *Df (2 L)TW12* is unrelated to the *btv* region, while *Tufted* (*Tft*) is a dominant bristle marker. A total of 5,966 flies (250 pair matings) were screened and 9 male recombinants recovered (Supplementary Table S1), giving a recombination rate of 0.15%.

To recover putative deletions between the BG01771 insertion and the insertion sites of nearby P-element insertions KG02815 or KG08320, the BG01771 insertion was first crossed to flies containing the transposase source, *w^+^*; *Sp*/*CyO*; *P {Δ2-3} Sb*/*TM6*, *Ubx*. Resulting *w^+^/w; BG01771/Sp; +/P {Δ2-3} Sb* females were crossed to either *w^+^/Y; KG02815* or *w^+^/Y; KG08320* males. Dysgenic *w/Y; BG01771/KG; +/P {Δ2-3}* males were crossed to *w*; *Sco*/*CyO* females and the offspring screened for white eyes. About 330 putative deletions were crossed to *w*; *btv^1^* 40AG13/*CyO* to test for btv phenotype.

### Behavioral assay: drop zone assay

To measure sedentary behavior and associated loss of flight ability, we developed the drop zone assay (DZA; Supplementary Figure S2). A square plexiglass cover, with a hole in its center, was placed on top of a 4 liter glass beaker. Flies were introduced in groups of 10 through the hole into the beaker, observed for 1 min and assigned a score from 1 to 6 as follows. Score 1: the fly did not touch the beaker bottom but flew directly upwards and landed on the plexiglass ceiling. Score 2: the fly landed on the beaker wall and climbed to the top. Scores of 3, 4, 5, or 6: the fly fell to the beaker floor, and either climbed immediately more than halfway to the top (3), climbed less than mid-way to the top (4), did not climb, but wandering around the beaker floor (5), or remained at the landing position (6).

### Electrophysiology

Extracellular recordings used to assay the sound-evoked potentials (SEPs) in the antennal nerve were performed as previously described (Eberl et al., 2000; Eberl and Kernan, 2011). Transepithelial potentials (TEP) and mechanoreceptor potentials (MRP) were recorded from adult anterior notopleural bristle organs essentially as previously described (Kernan et al., 1994), except that decapitated flies were mounted on a chlorided silver pin, which served as the basal electrode; transepithelial potentials were recorded with an EPC7 amplifier (List Medical) in current-clamp mode, and data was acquired and analysed with Powerlab/LabChart (AD Instruments). TEP values are relative to a zero obtained by inserting the bristle electrode into the body cavity, in cisepithelial configuration. The MRP was calculated as the maximal absolute change in TEP within 100 ms of the stimulus onset. Adaptation was calculated as the difference between the minimum TEP reached and the TEP averaged over the last 200 ms of a 1 s stimulus, as a percent of the MRP.

### Electron microscopy

*Drosophila* heads, with proboscii removed to facilitate infiltration, were fixed by immersion overnight at 4°C in a fixative containing 2.5% glutaraldehyde, 2.0% paraformaldehyde and 0.04% CaCl_2_ in 0.1 M phosphate buffer, pH 7.4 (PB). CaCl_2_ provides increased membrane stabilization. Heads were washed in PB, post-fixed with OsO_4_, dehydrated in an ethanol series and embedded in Polybed 812. Ultrathin sections (75 nm) were stained with aqueous uranyl acetate and lead citrate and examined with a Hitachi 7000 electron microscope.

### Fluorescent staining and imaging

The fluorescently-tagged IFT proteins IFT88 [GFP-NompB (Han et al., 2003)] and IFT140 [RempA-VenusYFP (Lee et al., 2008)], expressed from transgenes, were imaged *in situ* by their native fluorescence, in combination with immunolabelling of the proteins Futsch or NompA. Pupal antennae or pupal or pharate adult abdomen and halteres were dissected in PBT (0.2% Triton-X in PBS) and fixed in 4% formaldehyde in PBT. After three 10 min washes in PBT, specimens were incubated in blocking solution (PBT with 5% normal goat serum) for 1 h at room temperature. Futsch was labelled by incubating with mAb 22C10 (Developmental Studies Hybridoma Bank, Iowa City, IA) at a 1:100 dilution for 2.5 h at room temperature, followed by incubation with Alexa Fluor 546-conjugated goat anti-mouse antibody (Invitrogen) at 1:500 dilution, for 2 h, also at room temperature. NompA was labelled with rabbit anti-NompA (Chung et al., 2001) at a 1:500 dilution, followed by incubation with Alexa Fluor 647-conjugated goat anti-rabbit (Invitrogen) at 1:500 dilution. Specimens were mounted with Vectashield mounting media (Vectorlabs, CA) on a microscope slide and examined with a confocal microscope (Leica SP5).

### Southern analysis, PCR, and sequencing

Genomic DNA was isolated from adults and digested with EcoRI, HindIII or XhoI, run on agarose gel, and transferred to Hybond membrane according to instructions provided with the membrane. Southern blot analysis was performed using DIG High Prime DNA Labeling and Detection Starter Kit I (Roche) according to manufacturer’s instructions. A dot blot was conducted to confirm labeling and determine the concentration of labeled probe.

Primers presented in figures and used in PCR reactions include:

Se13F: 5’-ACTTGTTATCGTCCAACACC-3′.

Dhc9R: 5’-GTGCCAGCAGAACTTGATGA-3′.

PS5F: 5’-CAGCAACATCATCTGCAGCA-3′.

PS7R: 5’-ATAAGAATGCGGCCGCAATCTACAGGCGAC-3′.

## Results

### The *btv^1^* mutation strongly reduces hearing and affects chordotonal ciliary structure

In a behavioral mutagenesis screen (Eberl et al., 1997), we isolated a mutant, *5P1*, with reduced courtship song response. This mutant showed severely disrupted sound-evoked potentials (SEPs) in the antennal nerve (Eberl et al., 2000) and the corresponding gene was named *beethoven* (*btv*), with the *btv^5P1^* allele also referred to as *btv^1^* (Caldwell et al., 2003). Most *btv^1^* mutant flies retained a small SEP, especially if subjected to loud sound (Eberl et al., 2000), consistent with retention of a residual behavioral response to courtship song presentation (Eberl et al., 1997). This appears to be the null phenotype because overlapping deletions that remove the *btv* locus (such as *Df (2 L)TW119/Df (2 L)TW201*) also retain this residual SEP. To better understand the mutant phenotype, we examined the ciliary structure in more detail, and tested whether the residual response represented degeneration in progress. Thus, we compared electrophysiological phenotypes of *btv* mutants to controls 1–3 days after eclosion, after 9 days, and after 30 days. As *btv^+^* control flies, we used the genetic background on which the *btv^1^* mutation was induced (Table 2; Eberl et al., 1997). This strain, like the Canton-S wild-type strain, shows a general age-dependent decline in SEPs (Supplementary Figure S1) though little change before 30 days of age.

**Figure 1:**
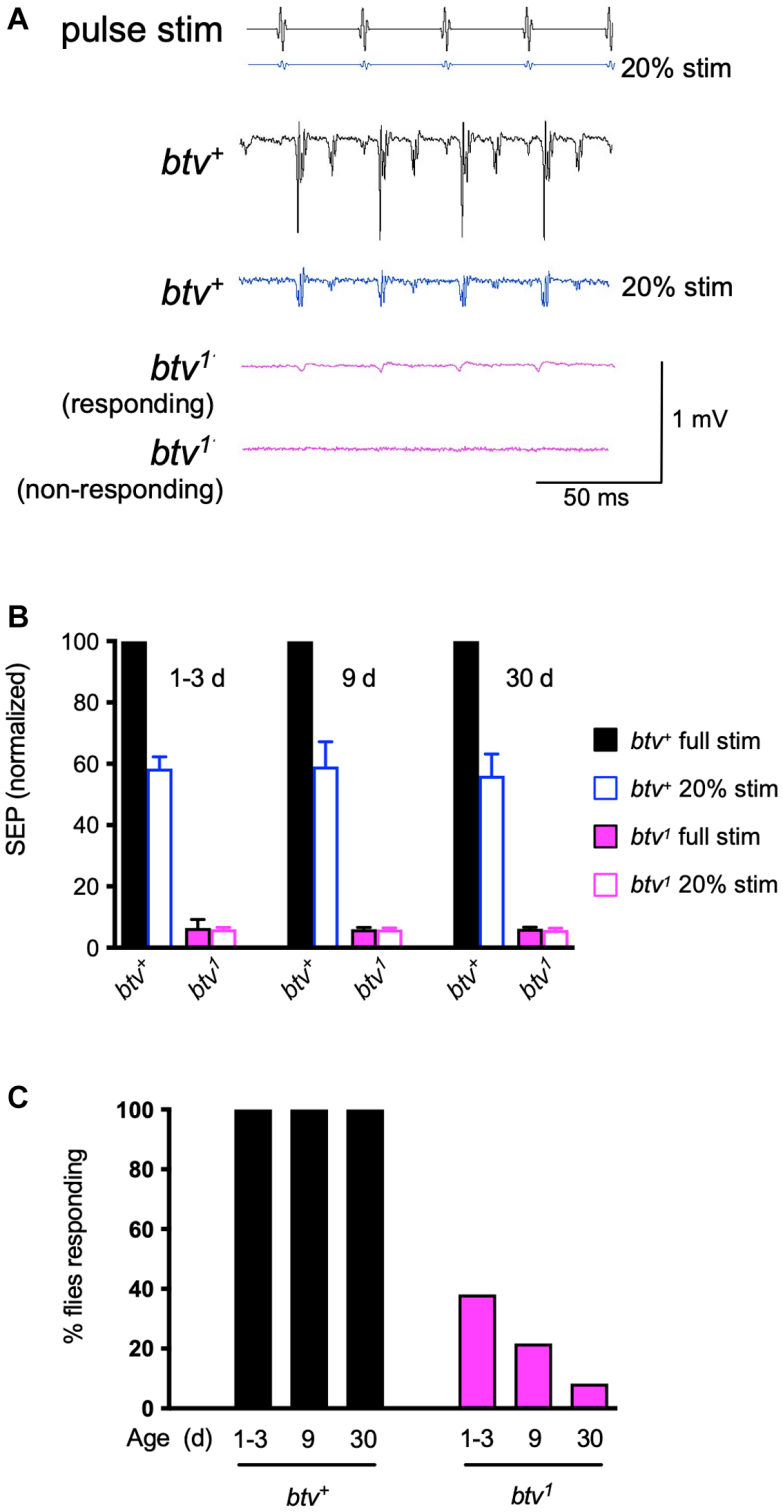
Electrophysiological phenotypes of *btv* mutant. **(A)** Sound-evoked potentials (SEPs) recorded from the antennal nerve, in response to near-field presentation of computer-generated sound stimulus (pulse stim) with 35 ms inter-pulse interval. The 40AG13 control strain (*btv^+^*) shows robust SEP (~1 mV) responses to the stimulus pulses at standard intensity. With the electrode position unchanged, presenting the same stimulus at lower intensity (20%, blue stimulus) still evokes a recognizable response with lower amplitude (blue response trace). Responses of *btv^1^* flies are somewhat variable, with some antennae showing a detectable, though low amplitude response (responding), and some antennae, particularly in older flies, showing no response (non-responding). **(B)** Quantitative SEP analysis with age. Filled bars represent responses to the standard sound intensity stimulus; open bars represent responses to the 5-fold lower stimulus amplitude. For normalization, the mean for *btv^+^* at normal intensity was set at 100% for each age group. The number of antennae recorded at 1–3, 9, and 30 days were 42, 16, and 21, respectively, for *btv^+^*, and 27, 25, and 36 for *btv^1^*. **(C)** Refer to key in panel **(B)**. While the mean SEPs appear very similar between the normal and 5-fold reduced stimulus in *btv^1^* flies and over the three age groups **(B)**, the percent of *btv^1^* flies with a recognizable response (see **A**) decreases with age.

Consistent with our previous results, we found that many, but not all, *btv^1^* flies showed small SEPs in response to a standard pulse song stimulus (Figure 1A). At a lower (20%) stimulus intensity, control flies showed SEPs of about 55% of their response to the standard amplitude regardless of age up to 30 days (Figures 1A,B). In contrast, average SEPs of *btv^1^* mutants were the same at loud and soft stimuli (Figure 1B). Therefore, we scored the percent of antennae that showed any detectable response. Compared to control flies, in which all antennae showed evoked responses, less than 40% of *btv^1^* antennae showed a response at 1–3 days, declining to less than 10% by 30 days (Figure 1C). Thus, the residual auditory function seen in *btv^1^* flies undergoes an early age-dependent decline.

**Figure 2:**
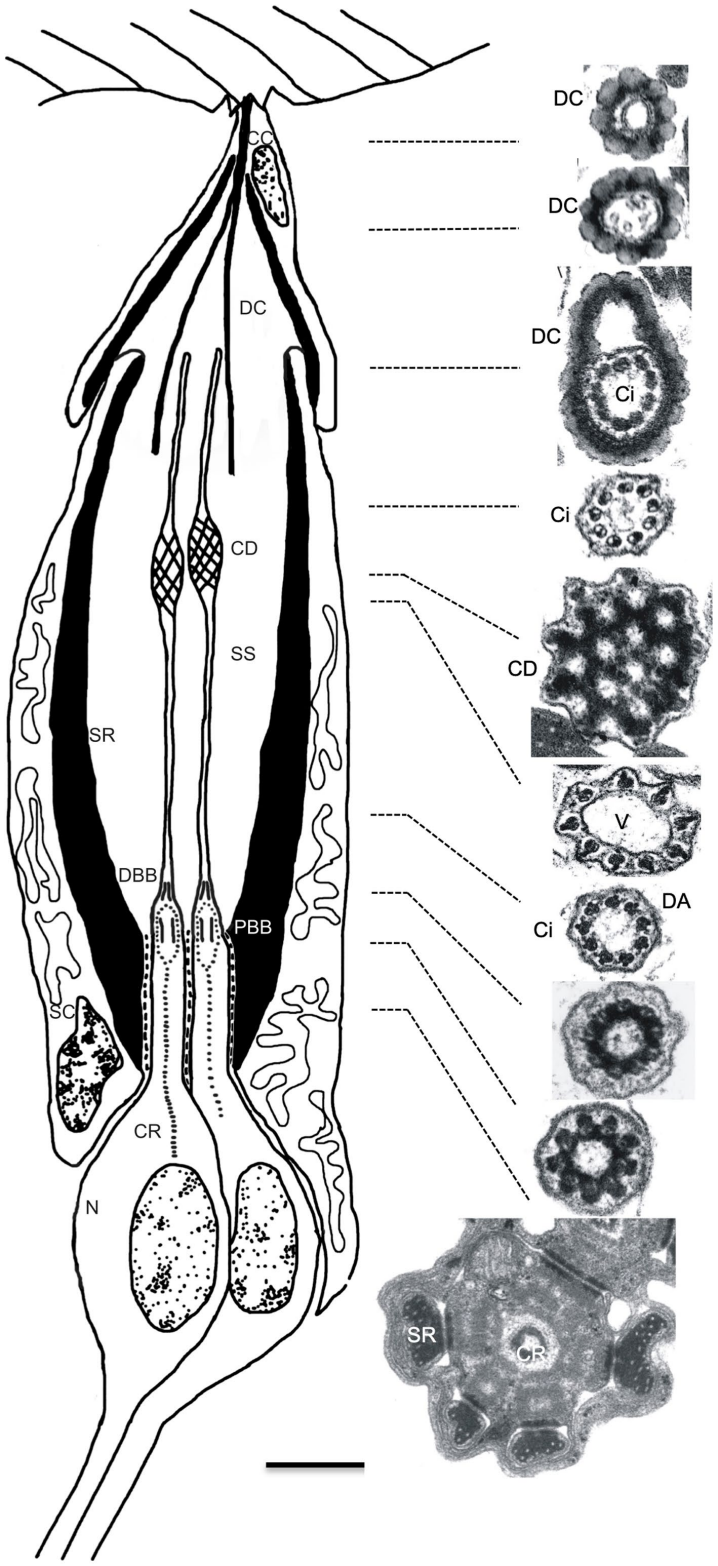
Structure of Johnston’s organ scolopidium in the wild type. Diagram (left) shows the cellular composition of a JO scolopidium, with apical attachment to the a2/a3 joint cuticle (top). The scolopale cell (SC) tightly envelops dendritic ends of neuronal cells (N), forming a scolopale space (SS) thought to contain a specialized receptor lymph. Apically, the cap cell (CC) surrounds the dendritic cap (DC) and seals the distal end of the scolopale space. Both the scolopale cell and the cap cell contain thick bundles of actin, called scolopale rods (SR), which form around a microtubule core. The neuronal dendrites are sensory cilia that grow out from the centriole-derived basal bodies [proximal and distal basal bodies (PBB and DBB)] and show long prominent ciliary roots (CR) that usually reach to the soma, and sometimes beyond into the axon. The sensory cilium (Ci) is subdivided into proximal and distal segments by the ciliary dilation (CD). Electron micrographs show cross-sections of the dendritic cap and a single sensory dendrite at the approximate levels indicated by the dotted lines. In sequence from the top, the electron micrographs on the right side show the beaded appearance of the dendritic cap (top 3 images) and variations in appearance of the dendrite. Within the dendritic cap, the distal cilium is closely associated with the cap material. The ciliary dilation (5th image from top) shows the expanded ciliary diameter, inclusion of an electron-dense matrix, and 9 peripheral microtubule doublets, which spread to continue around the dilation. Immediately below the dilation, and sometimes above as well, a vacuole space (V) appears as the microtubule doublets taper down to the normal ciliary diameter (6th image). Axonemal dynein arms (DA) are present in the ciliary segment below the ciliary dilation, especially visible in the 7th image, but not in the segment above the dilation (4th image). The bottom 3 images show the cross-sectional appearance proximal to the ciliary axoneme. The upper of these 3 images is at the transition from the distal basal body to the cilium, while the middle image depicts the proximal ciliary transition zone. The lowest image shows the inner dendritic segment with the central ciliary root. Scale bar (0.5 μm) applies to all electron micrographs. Diagram is not drawn to scale; the dendritic cap and cap cell are several times the length of the sensory cilia, but are shortened here for clarity.

**Figure 3:**
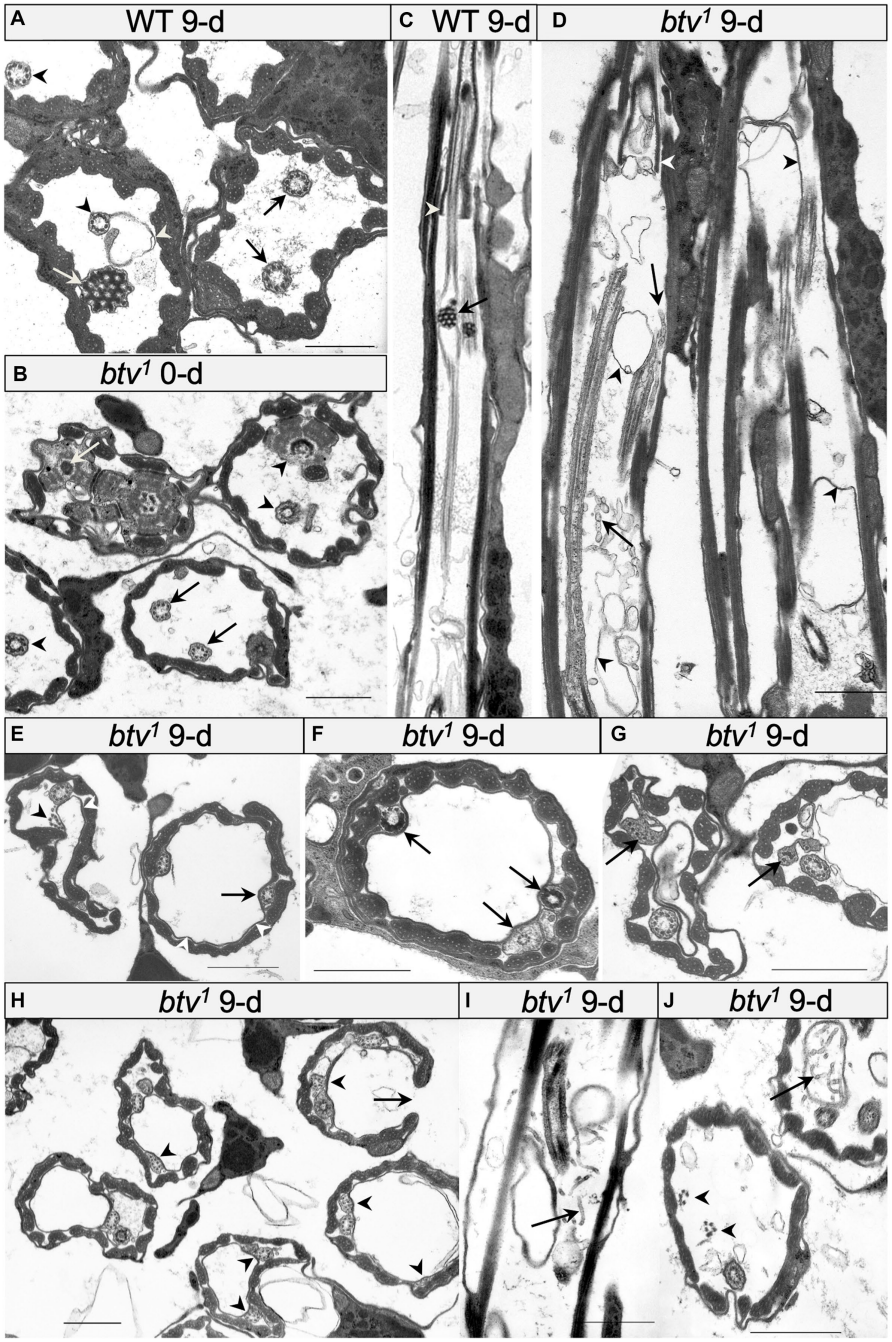
Morphological defects in *btv* and control JO scolopidia. **(A)** Cross-section through JO scolopidia of 9 days-old wild-type fly shows typical electron-dense ciliary dilation (white arrow), cilia bearing dynein arms (black arrows) proximal to the ciliary dilation, and cilia lacking dynein arms (black arrowheads) distal to the ciliary dilation. A small amount of excess membrane (white arrowhead) can sometimes be seen in the wild type. **(B)** Cross-section through proximal JO scolopidia of newly eclosed *btv^1^* fly shows many normal inner dendritic structures including ciliary rootlet (white arrow), and basal bodies (black arrowheads) but the proximal axonemal segments (black arrows) sometimes show fewer than 9 microtubule doublets. **(C)** Longitudinal section through *btv^+^* control scolopidium. In the proximal region of the cilium, the section grazes the cilium, but distally, the ciliary dilation with electron-dense matrix (black arrow) is visible, along with a grazing section nearby through the ciliary dilation of the second neuron. Beyond the ciliary dilation, the cilium is enclosed by the dendritic cap (white arrowhead). **(D)** Longitudinal section through two 9-day-old *btv* mutant scolopidia. Variable ciliary assembly is revealed by segments of these cilia. Some ciliary material is associated with the dendritic cap (white arrowhead). Extra membranous material (black arrowheads) appears in the scolopale space, and some fragmented material (black arrow) that may be degenerating axonemes, appears as “sausage-like” structure. **(E**–**G)** Cross-sections at mid-scolopale level of 9 days-old *btv^1^* JO. Excess membranes fill the scolopale space (white arrowheads), often pushing the cilia to the edge. Axonemes show a variety of disruptions (black arrowheads) such as lack of enclosing ciliary membrane, fewer than 9 microtubule doublets, unusual electron-dense inclusions or more severe ciliary disruptions. **(H)** Cross-sections through a 9 days-old *btv^1^* JO at mid-scolopale level, showing scolopale disruptions that include occasional crescent-shaped scolopale profiles (arrows). Many axonemes are missing microtubule doublets (arrowheads). (**I,J**) Longitudinal **(I)** and cross-sections **(J)** of 9 days-old *btv^1^* JOs show abnormal accumulations of tubular material (arrows). The origin of this “sausage-like” material is unknown, but may represent degenerating axonemal derivatives. A fragmented axoneme (arrowheads) is seen lacking a ciliary membrane. All scale bars are 1 μm.

**Figure 4:**
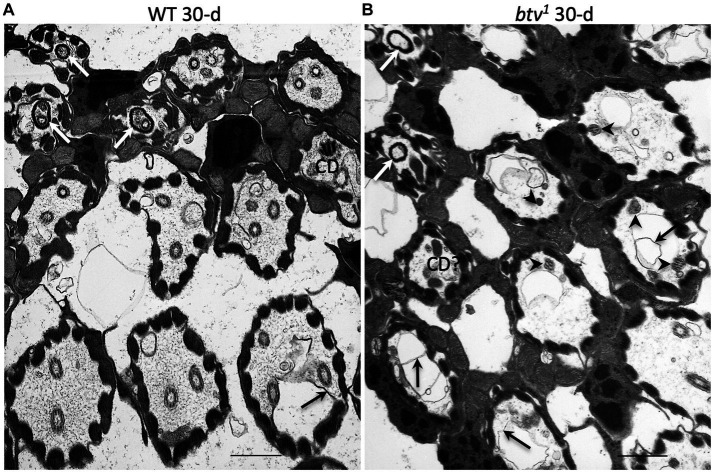
JO scolopidial structure in 30 days-old control and *btv* mutants. Slightly oblique sections through fields of JO scolopidia reveal structure at several levels from mid-scolopale (bottom right) to dendritic cap level (top left) in a control fly **(A)** and a *btv^1^* mutant **(B)**, both 30 days old. **(A)** The control scolopidia each show two or three sensory cilia within the scolopale space. One ciliary dilation (CD) is sectioned through its electron dense core, and three dendritic caps (white arrows) are seen in the upper left, enclosing ciliary tips. While some excess membrane (black arrow) is seen occasionally, ciliary integrity is maintained. **(B)** The *btv^1^* mutant at 30 days shows strong ciliary defects above the basal body level. Most scolopidia have only fragmented axonemes (arrowheads), if any. Some electron dense material that may represent degenerate ciliary dilations can be seen in some distal scolopale spaces (CD?), and beyond that, the dendritic caps (white arrows) appear mostly empty or enclosing only amorphous material. Scale bars: 1 μm.

To investigate in more detail the morphological effects on JO scolopidia resulting from loss of *btv* function, and to examine the degenerative effects at the morphological level, we used TEM to characterize JO scolopidia from control and mutant flies at 1–3 days, 9 days and 30 days old (Figures 2–4). The normal structure of an individual JO scolopidium is diagrammed in Figure 2, with TEM sections showing the inner and outer dendritic segments of the neuron, including the ciliary rootlet, the basal bodies, the proximal ciliary segment (bearing dynein arms), the ciliary dilation and the distal ciliary segment (lacking dynein arms). The scolopale cell, supported by actin-rich scolopale rods with embedded microtubules, encloses the sensory cilia within an extracellular cavity called the scolopale space, which likely contains a specialized receptor lymph to drive receptor potentials during mechanotransduction. The scolopale cell secretes NompA protein (Chung et al., 2001) into the extracellular matrix to form the dendritic cap, which connects the neuronal ciliary tips to the a2/a3 joint cuticle. A cap cell surrounds the distal end of the scolopale cell and enwraps the dendritic cap, but it is unclear whether it contributes proteins to the dendritic cap structure.

In the *btv^1^* mutant, we saw no significant changes to the inner dendritic segment at any of the ages tested, compared to control. The inner dendritic segments are tightly associated by desmosomes, both with each other and with scolopale rods in the apposed scolopale cell, and the base of the cilium always contains proximal and distal basal bodies and a ciliary root (Figure 3B). Near the basal bodies, the axoneme is usually complete with the expected nine microtubule doublets (Figure 3B). Beyond the ciliary base, the sensory cilia of *btv^1^* mutants show some segments constructed reasonably well, while other segments are fragmented and disorganized (Figure 3D) compared to *btv^+^* controls (Figures 3A,C). At the mid-scolopale level of *btv* JO at all ages examined, we found frequent loss of microtubule doublets and loss of ciliary membrane (Figures 3E–J), often resulting in very deformed ciliary profiles (Figures 3G,J). Despite these defects, we often see clear dynein arms present on the axonemes (Figure 3G). At the expected location of the ciliary dilation, about three fourths of the distance from the basal bodies to ciliary tip (Figure 4A), we never see the organized grid-shaped matrix of the ciliary dilation in *btv* flies. Instead, we sometimes observe a complex of additional membranes, vacuoles and microtubules embedded in electron dense material (Figure 4B).

We were surprised to find, in control animals, excess membranes accumulating in the scolopale space with age. The appearance of these membranes varies from scolopidium to scolopidium, from loosely organized membranous material floating in the scolopale space (Figure 4A) to robust vacuoles whose expansion occludes the cilia to the edge of the space, as in *btv* (Figure 3E). To ensure that this is not specific to our control strain, 40AG13, we checked the Canton S wild-type strain and found similar levels of membrane accumulation in the scolopale space with age (not shown). These membranes are similar in *btv* mutants and control flies; however, in *btv* flies 9 days and older, the ciliary occlusions are sometimes accompanied by deformation of the ring of scolopale rods, and even invagination of membranes between scolopale rods, resulting in crescent-shaped scolopidial profiles (Figure 3H). In addition, only in the *btv* mutants, starting at the 9 day time-point, we begin to see additional elongated tubular structures (Figures 3D,I,J) that resemble strings of sausages. These accumulations may correlate with fewer ciliary profiles at 30 days (Figure 4B), and therefore may represent degenerating ciliary material. Alternatively, the appearance of membranous vesicles in the scolopale space may be caused by ciliary vesicle shedding (Ojeda Naharros and Nachury, 2022), which could be enhanced upon loss of retrograde IFT. The effects of age on control and mutant scolopidia are summarized in Table 2. To test whether the degenerative effects could result from activation of apoptosis, we stained sections of 14 day old *btv* mutant and control antennae with the TUNEL technique, and found no difference in labeling (not shown), suggesting that the consequences of age on *btv* mutants are not apoptotic in origin.

**Table 2:** Summary of age effects on control and *btv* mutant JO scolopidial structure.

Feature	Control		*btv^1^*	
	Young	Old	Young	Old
1. Ciliary dilation	+	+	−	−
2. 9 × 2 arrangement of microtubule doublets	+	+	−	−
3. Cap structure	+	+	−	−
4. Additional membranes	+	+/−	−	−
5. Membrane loops at ciliary dilation	+	+	−	−
6. Scolopale invaginations	+	+/−	−	−
7. “Sausage-like” structure	+	+	+	−

For each phenotype, “+” indicates normal; “−” indicates defective; “+/−” indicates a mix of normal and defective scolopidia; “--” indicates strongly defective. “Young” refers to the phenotype of 1–3 day old flies, while “old” refers to 30 days old flies.

### Isolation and mapping of *beethoven* mutations

**Figure 5:**
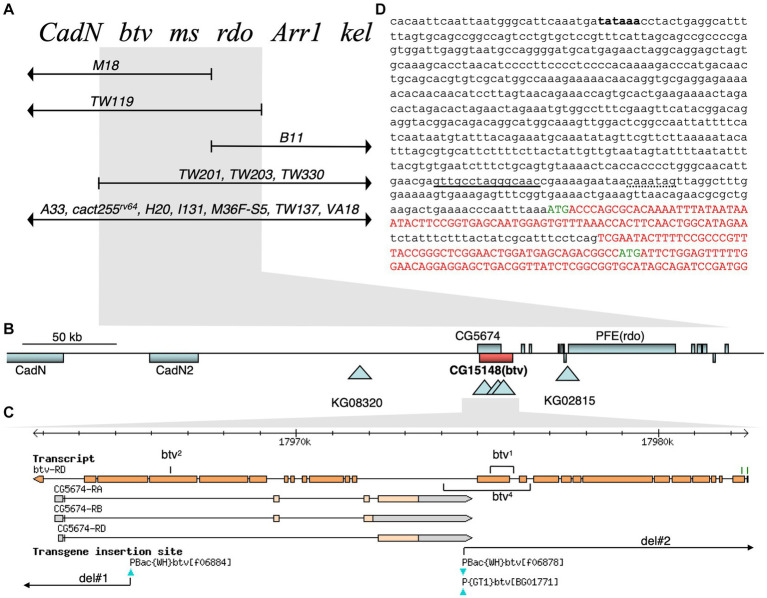
Genetic and molecular *btv* gene organization. **(A)** Complementation map of the *btv* region. Known genetic loci at the top are ordered by complementation with deletion chromosomes shown below. Deleted segments are shown by lines with arrowheads; deletion endpoints within the region, by vertical bars. Deletions showing the same complementation pattern are listed on the same line. Flies carrying the overlapping deletions *TW119* and *TW201* are viable, show the btv and rdo phenotypes, and are male sterile. The *btv* mutation is rescued by *Dp (2; Y)H3* (not shown). **(B)** Physical map of *btv* genomic region. Gene spans are shown as rectangles: genes above the line are transcribed to the right; those below, to the left. Triangles indicate transposon insertion sites. **(C)** JBrowse view of the *btv* gene, with exons represented by orange rectangles. Two putative *btv* translation start sites are indicated as green tick marks. All exons of the oppositely transcribed gene *CG5674* are fully nested within the *btv* introns. Transposon insertions are indicated below. Positions of the small deletions in *btv^1^* and *btv^4^* are indicated, as are the FRT-mediated deletions (*del#1* and *del#2*). **(D)** Genomic sequence at 5′ end of *btv* gene. Putative coding sequences are shown in upper case red or green letters, with green letters indicating the two putative translation starts. The 29 base pair intron between exons 1 and 2 has not been experimentally confirmed. A perfect consensus X-box sequence for Rfx transcription factor binding is underlined, and a consensus F-box sequence for Fd3F transcription factor binding is shown with dotted underline (Newton et al., 2012). A putative upstream TATA box sequence is in bold font but has not been functionally confirmed.

To understand the molecular basis of the btv phenotype, we pursued identification of the *btv* gene. Therefore, we carried out a series of genetic mapping studies and isolated several new alleles. As we shall describe, *btv* is identical with *Dhc36D* (also called *DHC1b* and *CG15148*), and encodes the retrograde IFT dynein motor, Dync2h1. Henceforth, we will use *Dync2h1*, according to the nomenclature proposed by Braschi et al. (2022). Because the *Dync2h1* gene is strongly mutually nested with the *CG5674* gene, it was critical that we used multiple approaches to conclusively determine whether *btv* corresponds to *Dync2h1*. This analysis is summarized below and in Figure 5 and described in additional detail in the Supplementary Material.

First, we mapped the *btv^1^* allele isolated in an ethyl methanesulfonate (EMS) mutagenesis screen (Eberl et al., 1997) to the 36E1-3 polytene chromosome region to the left of the *rdo* locus by deficiency mapping (Eberl et al., 2000; Caldwell et al., 2007) (Figure 5). Using *P*-induced male recombination (PIMR) (Chen et al., 1998), we refined the *btv* map position to the right of the *KG08320 P*-element (Figure 5 and Supplementary Table S1). Next, while the *P {GT1}BG01771 and PBac {WH}f06878* insertions (Figure 5C) do not show a btv phenotype, *btv^5^*, the *PBac {WH}f06884* insertion in exon 23 of *Dync2h1* (Figure 5C and Table 1), fails to complement other *btv* alleles. Using a variety of strategies (see details in Supplementary Material), we generated several additional mutations, including *btv^4^*, del#1, and del#2, which likely affected both *Dync2h1* and *CG5674*.

**Figure 6:**
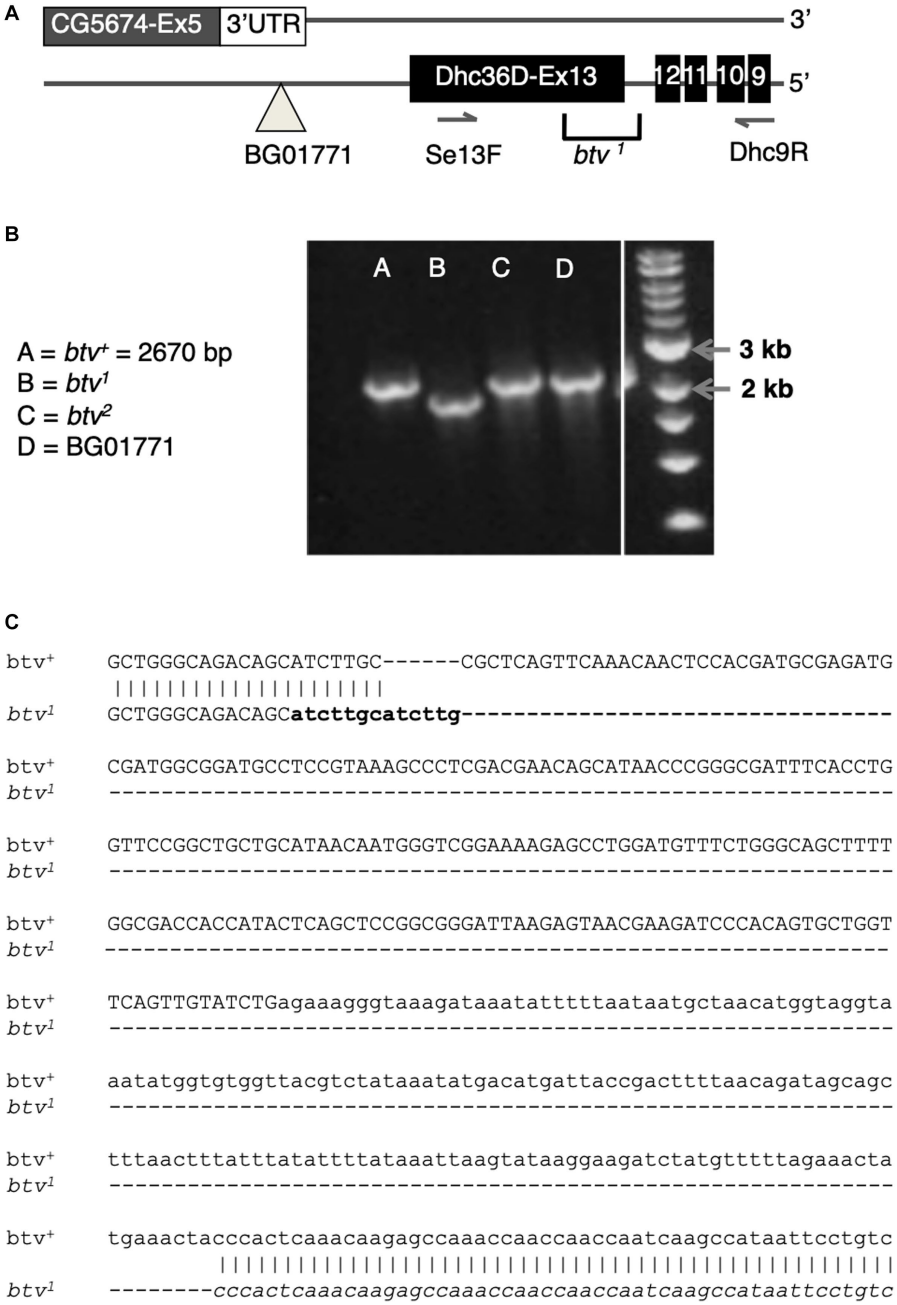
The *btv^1^* mutation is a small deletion that affects *CG15148* but not *CG5674*. **(A)** Diagram of the region near *CG15148* exon 13 encoding Dync2h1 (Dhc36D). The BG01771 transposon insertion site and the positions of primers used in B are shown. **(B)** PCR results with genomic DNA templates listed. The expected 2,670 bp amplicon is recovered in all genotypes shown except *btv^1^*. **(C)** Sequencing of the *btv^1^* genomic DNA in this region confirms a small deletion of 401 bp, in addition to a 6 bp insertion that appears to be a tandem duplication of sequences (in bold) flanking one end of the deletion. Exon sequences are shown in upper case, intron in lower case.

Because the *btv^1^* mutation was generated by EMS, we expected to find a point mutation. Further characterization, however, revealed a 401 bp deletion along with a 6 bp insertion (Figure 6C). The deletion removes 125 bp of *Dync2h1* exon 13 and part of the intron between exons 12 and 13. Because this deleted region excludes any *CG5674* sequences, the *btv^1^* lesion firmly establishes *Dync2h1* as *btv*. This is supported by our discovery in *btv^2^*, using Mismatch Endonuclease Arrays (MENA) (Comeron et al., 2016), of a single nucleotide deletion in *Dync2h1* exon 22 resulting in a frameshift that introduces an early stop codon (Comeron et al., 2016). This position is in the middle of a large intron in the overlapping gene *CG5674* on the opposite strand, and therefore unlikely to affect its function (Figure 5C). Finally, we found numerous sequence polymorphisms in different lab strains compared to the sequence used for the genome annotation (Supplementary Table S1), emphasizing the need to compare sequence deviations relative to the background strain on which the mutation was induced.

We find no differences in the electrophysiological phenotypes between *btv* alleles that also disrupt *CG5674* and those that do not. Thus there is no evidence that *CG5674* contributes to the btv phenotype.

Taken together, all these observations demonstrate that the *btv* gene corresponds to *Dync2h1*.

### *Beethoven* encodes the retrograde IFT dynein motor

All our evidence indicates that *btv* corresponds to *CG15148*, which encodes a dynein heavy chain most similar to members of the Dync2h1 isoform. The annotated intron-exon structure and protein sequence evolved significantly over the course of this project. We sequenced PCR amplicons from cDNA of adult heads from 40AG13 and *w^1118^* control strains to define the intron-exon structure. Each amplicon was generated at least two independent times to distinguish sequence changes from PCR-induced changes. The current annotation (*D. melanogaster* r6.50; Gramates et al., 2022) resembles our empirical data most closely. Exons 2–24 (Figure 5C) match our cDNA sequencing results. We did not confirm exons 1 and 25, reported in the annotation. However, protein sequences predicted in exon 1 retain considerable conservation with Dync2h1 isoforms from other insects, and some modest conservation with vertebrate homologs. Upstream of the putative starting methionine in exon 1 (Figure 5D), we found a putative TATA box at about 635 bp upstream, and a perfect match to a Rfx transcription factor binding site consensus at about 100 bp upstream and to a Fd3F consensus site about 90 bp upstream (Laurençon et al., 2007; Cachero et al., 2011; Newton et al., 2012). This is consistent with Rfx-dependent (Laurençon et al., 2007) and Fd3F-dependent (Newton et al., 2012) regulation of *btv*. While the starting methionine in exon 1 is more likely correct, a second possible starting methionine is present in exon 2 (Figure 5D; Supplementary Figure S4). Protein sequences predicted from exon 25 share similarity with closely related insect homologs but not with vertebrate homologs. However, until the splicing structure is confirmed experimentally, we cannot be certain of the structure at the C-terminus. Assuming the longest prediction, the protein would consist of 4,237 amino acids, for a predicted molecular weight of 481.45 kDa, barring post-translational modifications (Supplementary Figure S4). Consistent with the structure of other dynein heavy chains, this Dync2h1 contains 4 conserved ATP-binding domains, called P-loops (Holzbaur and Vallee, 1994), as highlighted in the predicted protein sequence (Supplementary Figure S4) and in alignment with the Dync2h1 sequences from several species across the ciliated taxa (Supplementary Figure S5). The sequence of P-loop 1, GPAGTGKT, is identical to that in all other dyneins. P-loops 2 and 3 show intermediate levels of conservation, while P-loop 4 shows the least similarity between organisms (Supplementary Figure S5). To examine the localization of the Btv protein, we generated a monoclonal antibody against a Btv peptide in collaboration with the Developmental Studies Hybridoma Bank. Unfortunately, this antibody showed no signal on Western blots or in tissues (data not shown).

### *Beethoven* mutation disrupts retrograde IFT in chordotonal sensory cilia

To confirm that this Dync2h1 dynein motor performs the retrograde IFT function in *Drosophila*, we examined the morphological phenotype of sensory neurons in the auditory organ, JO. We previously showed that the kinesin II motor, including the Klp64D heavy chain and DmKAP, mediates the anterograde IFT motor function (Sarpal et al., 2003). Loss of anterograde function causes complete failure to extend an axoneme from the JO basal bodies. With loss of retrograde motor function, we expect anterograde transport to be largely intact, resulting in partial axoneme assembly. However, retrograde transport is required for clearing the growing cilium of assembly byproducts, so we expect that as growth and assembly proceed, proteins and complexes that would normally be cleared will accumulate, leading to impaired assembly. Consistent with this, we previously reported morphological defects that include partial and variable assembly of the axoneme and abnormal swellings of the ciliary membrane (Eberl et al., 2000). Our further ultrastructural analysis (Figures 2–4) shows ciliary defects that are fully consistent with loss of retrograde IFT.

**Figure 7:**
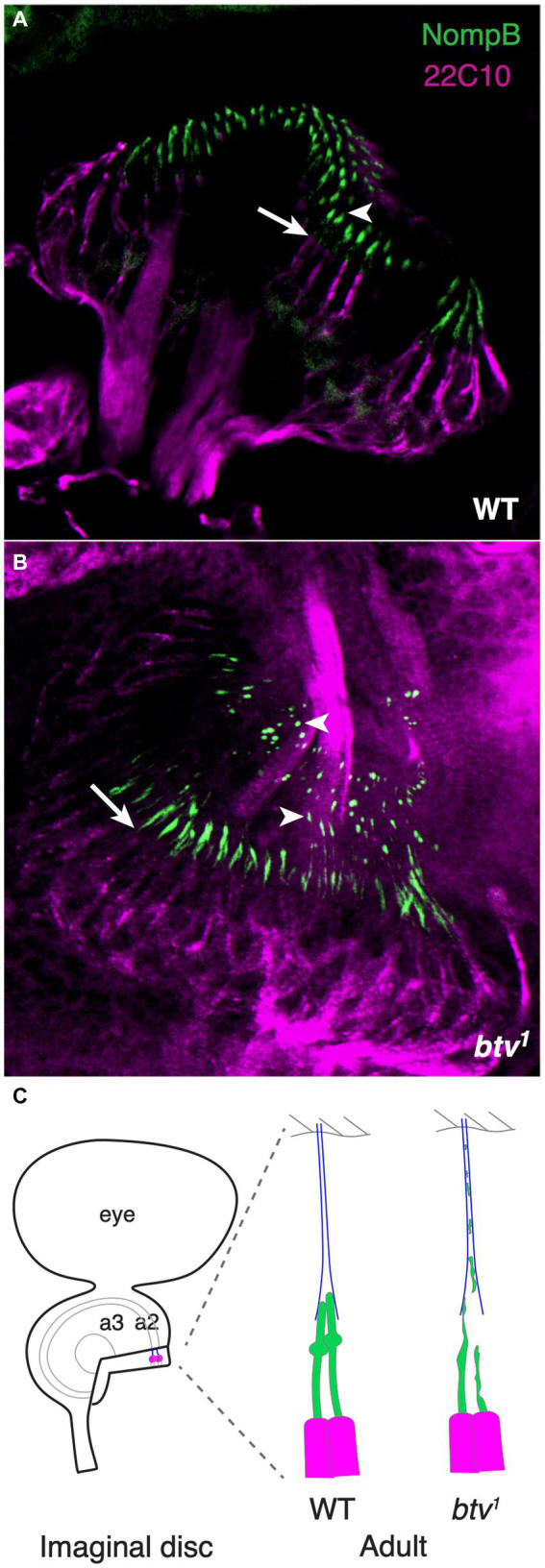
NompB is delocalized in *btv* mutant chordotonal neurons. **(A)** GFP-tagged NompB (green), encoding the IFT-B protein IFT88, normally localizes in the ciliated outer dendritic segment distal to the basal bodies at the end of the inner dendritic segment (arrow indicates transition from inner to outer dendritic segment) in the late pupal JO, ending at the ciliary tip (arrowhead) just distal to the ciliary dilation. Counterstain is mAb 22C10 (magenta), which stains all neurons, and labels chordotonal neurons including the inner dendritic segment, but not the cilium. **(B)** In JO of late pupal *btv^1^* mutants, GFP-NompB is delocalized beyond the basal bodies (arrow), with some GFP-NompB protein delocalized to regions extending along the dendritic cap (arrowheads), in some cases as far as the a2/a3 joint. **(C)** The schematics highlight our interpretation of the normal and mutant localization of NompB. JO development from newly specified sense organs in the imaginal disc involved extensive elongation enroute to the adult configuration, with the tubular dendritic cap (blue) maintaining the physical connection between the cilia tips and the antennal joint cuticle. Structural defects in *btv* mutant cilia results in loss of ciliary fragments, leaving a developmental “trail of crumbs”.

If retrograde IFT is disrupted, we should expect accumulation of IFT components in the cilium due to failure of the retrograde transport clearing function. Thus, we examined the expression and distribution of the GFP-NompB protein (Han et al., 2003), which encodes the IFT complex B protein homologous to Chlamydomonas IFT88, in *btv* mutants. In the mature JO, GFP-NompB normally localizes to the cilium, with most fluorescence near the ciliary tip, in the vicinity of the ciliary dilation (Figure 7A). In JO cilia of similarly staged in *btv^1^* mutants, imaged under identical conditions, the GFP-NompB protein correctly localizes to the cilium. However, localization within the cilium is disrupted, showing excess fluorescence both in proximal cilium close to the basal bodies, as well as ectopic fluorescence distally (Figure 7B). This distal fluorescence likely represents ciliary material sloughed off in a “trail of crumbs” fashion along the length of the tubular dendritic cap, where it is left behind developmentally rather than being cleared toward the cell body by retrograde IFT motor activity. Unlike growth of unattached cilia such as *Chlamydomonas* flagella, the JO cilia, embraced by the dendritic cap, extend in concert with extensive developmental stretching of the scolopidium (Figure 7C). The dendritic cap forms early in antennal development (Todi et al., 2008), and in the *btv* mutant, developmental stretching culminates in the cilium stretching to its normal length despite failure of proper axonemal growth. Thus, finding distal fragments of cilium indicates a failure of the cilium to remain intact during scolopidium elongation, rather than excess extension of the cilium. Therefore, the delocalization of GFP-NompB in *btv* mutants supports a retrograde IFT transport function for the *btv*-encoded dynein.

### *Beethoven* mutation de-localizes IFT140 and reduces mechanoreceptor potentials in external sensory organ dendrites

**Figure 8:**
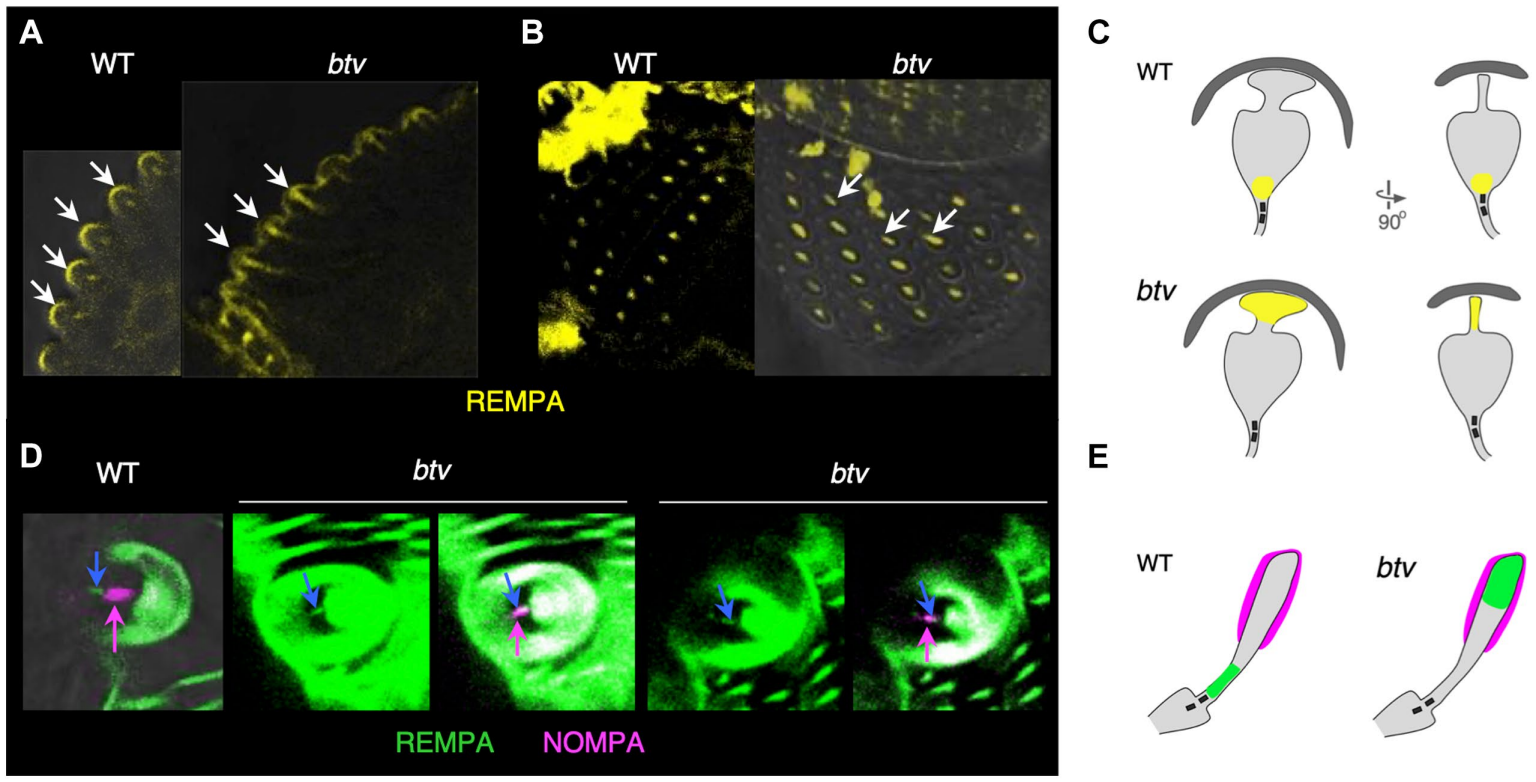
IFT140/RempA is mislocalized in *btv* mutant es organs. Transverse **(A)** and overhead **(B)** views of haltere campaniform sensilla in pharate adults expressing YFP-labelled IFT140 (RempA). Arrows in **(A)** indicate the apex of autofluorescent campaniform domes, highlighting in the *btv* mutant abnormal extension of IFT140 labeling beyond the connecting cilium where it is normally restricted, into the distal non-axonemal region of the dendrite. Arrows in **(B)** indicate elongated IFT140 staining in *btv* mutant dendrites. **(C)** Orthogonal sketches of the ciliary outer segments, based on Figure 1 of Sun et al. (2019). IFT140 is localized at the connecting cilium in the wild type, but is mislocalized to the blade-shaped distal tip in *btv* mutants, appearing as a line instead of a dot in the overhead view. **(D)** Bristle bases in the pharate adult abdomen, in which the dendritic cap that covers the distal outer segment is labelled with NompA (magenta). **(E)** Schematic of the outer segment, drawn after Figure 1 in Walker et al. (2000). In the wild type, IFT140 (blue arrow) localizes proximal to the dendritic cap (magenta arrow), but in *btv* mutants, the two proteins are colocalized. Two examples of mutant abdominal bristles are shown; for each, the left panel shows the RempA channel alone, while the right panel shows both IFT140/RempA and NompA channels.

Cachero et al. (2011) reported that Dync2h1 transcripts in embryos show a “chordotonal-enriched pattern,” with some additional lower level expression in es organs. To test whether btv mutant phenotypes are confined to ch organs, we examined the localization of RempA-YFP, a labelled version of the IFT140 protein (Lee et al., 2008) in *btv* mutant campaniform organs and bristle organs. In ch organs, we had previously shown that RempA-YFP is de-localized in *btv^1^* mutants, extending beyond the normal location of the ciliary tip (Lee et al., 2008). In campaniform organs of the haltere, RempA-YFP is normally confined near the proximal connecting cilium and proximal to the distal, non-axonemal region of the dendrite, but in *btv* mutants RempA is released distally, extending to the dome and filling the blade-shaped distal tip of the dendrite (Figures 8A–C). Similarly, in *btv* mutant bristle organs of the adult abdomen, RempA colocalizes with the distal dendritic cap marker NompA, in contrast with their discrete localization patterns in the wild type (Figures 8D,E).

**Figure 9:**
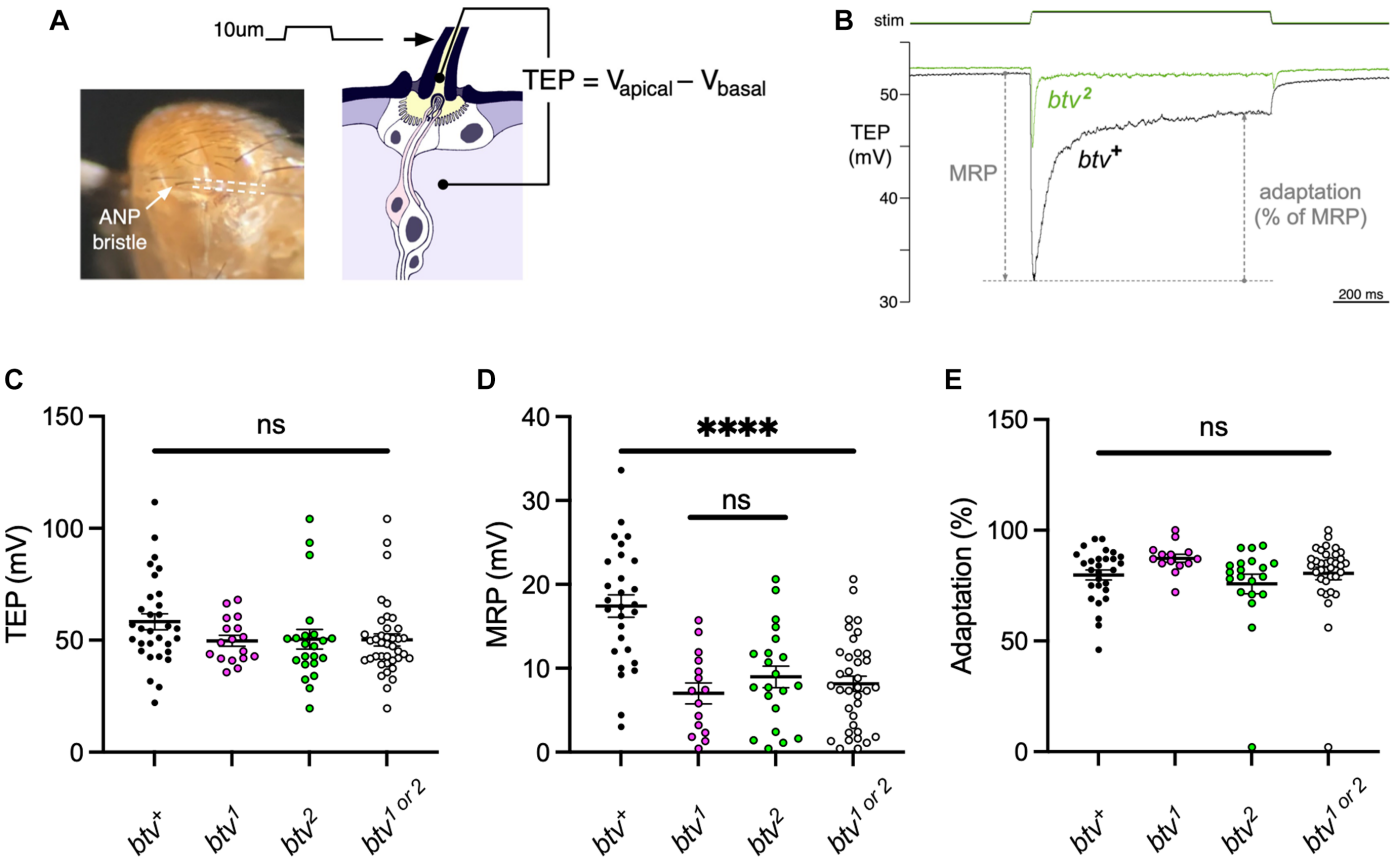
*btv* mutations affect mechanoreceptor potentials but not transepithelial potentials in bristle organs. **(A)** Recording bristle transepithelial potentials. The decapitated fly is mounted on a chlorided silver pin, which serves as a basal reference electrode. The anterior notopleural (ANP) bristle is cut to half its length, giving access to the lymph in apical extracellular space, and a saline-filled glass microelectrode (outlined by dashed lines), attached to a piezoelectric positioner, is placed over the cut end. The TEP, an apical-positive potential maintained by the ion pumping activity of bristle support cells, is recorded as the voltage difference between the apical and basal electrodes. **(B)** Representative mechanoreceptor potentials (MRP) recorded from *btv^+^* and *btv^2^* flies; each trace is an average of 5 trials. The MRP value is the maximal change in TEP immediately following a 10 μm step deflection of the microelectrode and bristle; adaptation is calculated as the % reduction in this change by the end of the 1 s stimulus. C-E: *btv* mutants show a significant reduction in MRP amplitudes, but no significant change in resting TEP or in adaptation. **(C)** TEP measured in *btv^+^*, *btv^1^*, and *btv^2^*. Each dot represents the TEP of a single bristle. Horizontal bars indicate means, error bars indicate SEM. The point scatter bar on the right includes the data from both *btv* alleles tested (*btv^1^* and *btv^2^*). TEP is unaffected in *btv* mutants compared, either separately or together, to *btv^+^*. **(D)** MRP is significantly reduced in *btv* mutants. Symbols as in **(C)**. **(E)** Adaptation is unaffected in *btv* mutants. Symbols as in **(C)**.

To test for electrophysiological effects of *btv* mutations on bristle organs, we recorded transepithelial potentials (TEPs) and mechanoreceptor potentials (MRPs) (Figures 9A,B) as previously described (Kernan et al., 1994). We found that, although TEPs do not differ significantly between *btv* mutants and control flies (Figure 9C), MRPs are reduced by about 50% in *btv* mutants (8.1 ± 0.9 mV) compared to controls (17.4 ± 1.3 mV; *t*-test with Welch’s correction, *p* < 0.0001) (Figure 9D). Adaptation of the MRP, (ratio of peak and end-stimulus MRP amplitudes) is the same in *btv* and controls (Figure 9E).

Regarding age effects on bristle organ function, although MRP amplitudes are slightly reduced in old (15.5 ± 1.7 mV) vs. young (20.9 ± 1.7 mV) control flies (*t*-test, *p* < 0.04) (Supplementary Figure S6), this is not significant in *btv* mutants (7.6 ± 1.6 mV vs. 8.5 ± 1.1 mV; *t*-test, *p* = 0.66). Six out of 38 old bristles were non-responders, but these were divided between both control (3 of 21) and *btv* mutants (3 of 17). These data do not support accelerated degeneration in *btv* mutant bristles.

## Discussion

### The Dync2h1 encoded by *btv* performs IFT in chordotonal organs

Several lines of evidence support the conclusion that the *btv*-encoded Dync2h1 carries out the retrograde IFT function. First, as in all other organisms characterized, there is only a single Dync2h1 gene in the *Drosophila* genome. Ciliary function for Dync2h1 is supported by studies that report *btv* gene expression in ciliated cells. The *btv* gene expression is regulated by the Rfx transcription factor (Laurençon et al., 2007), as would be expected for a central component of IFT. In that study, *btv* gene expression was reduced by 124 fold in *dRfx* mutants. By *in situ* hybridization, *btv* gene expression was seen in embryonic ch organs, and in embryonic cell-sorting microarray experiments, *btv* gene expression showed 8-fold enrichment in cell lineages associated with the chordotonal proneural gene *atonal* (Cachero et al., 2011). Furthermore, the *btv*-encoded Dync2h1 is directly regulated by the forkhead transcription factor Fd3F (Newton et al., 2012), a regulator of ciliary genes. Binding sites for these two transcription factors are identified in the promoter region of the DCH1b gene [Figure 5D, (Laurençon et al., 2007; Newton et al., 2012)].

Secondly, the profile of chordotonal defects we see in the *btv* mutant, including axonemal disruption and loss of ciliary integrity that worsens with distance from the basal bodies, is entirely consistent with loss of retrograde IFT. The defects are remarkably similar to those we documented for *rempA* mutants (Lee et al., 2008), encoding IFT140, an IFT-A particle protein that should be transported by Dync2h1 in retrograde IFT. This similarity is consistent with the idea that the Btv-mediated assembly of cilia is carried out through transport of IFT-A complexes including RempA.

Additional evidence supporting a retrograde IFT function for the Btv protein involves the mislocalization of other components in *btv* mutants. Here we show that NompB, encoding the IFT-B complex protein IFT88, is abnormally distributed in defective cilia of *btv* mutant JO. This agrees with the finding of Lee et al. (2010) that both IFT88 and the TRPN ion channel NompC are mislocalized in *btv* mutants. Also, in embryonic ch organs, Kwon et al. (2020) reported that the transport requirements of NompC and TRPV ion channel Iav for Dync2h1 are different, where Dync2h1 is required for both pre-ciliary trafficking and retrograde transport of NompC, primarily in the distal compartment, while Iav only requires Dync2h1 for the former. Furthermore, Lee et al. (2018) used time-lapse imaging to show that retrograde movement of GFP-NompB is absent in *btv^1^* mutants. NompC is normally localized only to the distal ciliary segment, beyond the ciliary dilation, and NompB and Iav are delimited to the proximal segment, but *btv* mutants lack a ciliary dilation and lose the sequestered localization of these proteins. Furthermore, we previously showed that RempA, normally restricted to the ciliary dilation by the adult stage, and the Iav, normally delimited to the proximal ciliary segment, are also mislocalized in *btv* mutants (Lee et al., 2008). Mutation of *btv* also results in redistribution of the Eys/Spam extracellular matrix protein that is normally deposited into the scolopale space, and that provides protection against desiccation at high temperature conditions (Cook et al., 2008). This protein is normally distributed into a major mid-scolopale space region and a minor proximal region immediately adjacent to the inner dendritic segment; in *btv* mutants this collapses into a single larger proximal cluster (Lee et al., 2008). It is not clear whether this redistribution arises from loss of a positioning cue provided by the sensory cilium, or whether distal ciliary bloating or membrane leakage forces the scolopale space material more proximally. On the other hand, the *Drosophila* Tubby-like protein dTulp is not mislocalized in *btv* mutants (Park et al., 2013). In summary, in the current study we show detailed ultrastructural evidence of the *btv* mutant, along with mislocalization of ciliary proteins in the *btv* mutant background, including NompB and RempA. Overall, the *btv* mutant morphological defects combined with the spectrum of mislocalized proteins demonstrate clearly that Dync2h1 encoded by *btv* is centrally involved in retrograde IFT in ch organs.

### Functional role of Dync2h1 encoded by *btv* in external sensory organs

Our finding of altered relative distribution of RempA-YFP and NompA in *btv* mutants is consistent with our finding of functional defects as reflected by reduced MRPs. Nevertheless, the functional consequences of *btv* dysfunction in es organs is relatively mild compared to those in ch organs. In es organs, the axonemal segment of the cilium is very short, while the tubular bundle of the distal dendrite is non-axonemal. In contrast to the defective ch cilia tips, the distinctive tip shape of campaniform sensilla is retained, but accumulates RempA (Figure 8). If Dync2h1 requires axonemal structure as a substrate to move cargo, then it may be possible for some cargoes to diffuse along such a short distance sufficiently well to move into the dendrite. Thus, despite de-localization of RempA and NompA, assembly of sensory structures in es organs is sufficient to provide some mechanosensory function. Even in ch organs, where the ciliary segments are relatively long, some residual sensory function is maintained in *btv* null mutants. Alternatively, it is possible that in es organs, the Dync2h1 function is not specifically IFT *per se*, but a related transport function for certain cargo proteins, including RempA. Dync2h1 does not appear to have a role in establishing the TEP, suggesting that the ion transport mechanisms to establish and maintain the receptor lymph properties do not depend on this dynein motor. Although some forms of mechanosensory adaptation are motor-driven, loss of Dync2h1 does not affect the MRP adaptation, suggesting that adaptation does not require this dynein.

### Use of IFT dynein in diverse cilia and flagella

Until recently, ciliary assembly was thought to be universally dependent upon IFT. Consistent with this, we find that retrograde IFT function mediated by Dync2h1 in *Drosophila* is essential for normal ciliary assembly in ch organ sensory cilia, including JO. In es organs, which have short ciliary segments, the Dync2h1 has a more modest requirement. Dync2h1 is dispensable in sperm flagella: *btv* mutants are male-fertile (Eberl et al., 2000). Other exceptions to IFT-dependence have been found. This supports the idea that in *Drosophila*, contrary to many other taxa, assembly of the sperm flagellum axoneme employs an IFT-independent mechanism (Han et al., 2003; Sarpal et al., 2003; Lee et al., 2008). While the Rfx transcription factor is transiently expressed during spermatid development (Vandaele et al., 2001), there is no evidence available that Rfx activates ciliary genes for spermiogenesis. Furthermore, in a survey of ciliary motility machinery, it was found that a surprising number of axonemal components are encoded by separate genes in sperm compared to mechanosensory cilia (Zur Lage et al., 2019). While IFT is highly conserved in *Drosophila*, it is activated only in sensory cilia, not in the male germline. In mammals, however, IFT is essential for spermiogenesis, though not for maintenance of sperm tail flagella (San Agustin et al., 2015).

In *Tetrahymena*, Dync2h1 (*dhc-2*) appears to be dispensable for ciliary integrity, though it regulates cilia length such that in mutants, ciliary length is variable and cilia are fewer in number (Rajagopalan et al., 2009). Furthermore, in this ciliate, IFT122A, an IFT-A complex protein, participates in retrograde trafficking of IFT88 and IFT172 proteins; these proteins accumulate in the cilia but ciliary assembly is not significantly disrupted (Tsao and Gorovsky, 2008). On the other hand, Dync2h1 phenotypes in *Chlamydomonas* (Pazour et al., 1999) and in *Caenorhabditis* (Signor et al., 1999; Wicks et al., 2000) are more severe, while the phenotypes we see in *Drosophila* ch organs are intermediate, allowing a small amount of residual sensory function. Thus, there is both taxonomic and cell type variation in the degree to which ciliary assembly can proceed with compromised retrograde IFT dynein function.

### Residual hearing function in *btv* mutants decreases with age through non-apoptotic degeneration

Our finding that *btv* mutants, while suffering strong hearing loss, are not completely deaf in contrast to other auditory mutants such as *tilB* (Kavlie et al., 2010), *iav* and *nan* (Kim et al., 2003; Gong et al., 2004) and the kinesin II mutants *klp64D* and *DmKAP* (Sarpal et al., 2003). This allowed us to test for progressive hearing loss, leading to our finding that by 30 days of age, *btv* mutant flies have a significant reduction in auditory capacity, evident primarily in the reduced fraction of flies retaining any residual auditory response. Morphologically, we see strong degeneration in many mutant cilia. However, we see no evidence that the sensory neurons die during this interval, and the lack of TUNEL labeling is consistent with degeneration and resorption of material only in the ciliary compartment.

Even in wild-type flies we see some hearing loss with age, consistent with other studies (Keder et al., 2020). We also see morphological changes with age that likely reflect the fact that, as in vertebrates, the auditory periphery encounters strong metabolic challenges. These changes include the deposition of excess membranes and increasing signs of putative disruption of ionic homeostasis. The morphological changes correlate with age-dependent reductions in hearing, but at present we cannot determine whether there is a causal relationship between these factors. The answer to this question must await further study, and may require the ability to record from single auditory neurons.

## Data availability statement

The datasets presented in this study can be found in online repositories. The names of the repository/repositories and accession number (s) can be found in the article/Supplementary material.

## Ethics statement

The manuscript presents research on animals that do not require ethical approval for their study.

## Author contributions

YS: Conceptualization, Data curation, Formal analysis, Investigation, Methodology, Visualization, Writing – original draft, Writing – review & editing. JJ: Conceptualization, Data curation, Formal analysis, Investigation, Methodology, Visualization, Writing – review & editing. ES-L: Conceptualization, Data curation, Formal analysis, Investigation, Methodology, Visualization, Writing – review & editing. EL: Conceptualization, Data curation, Formal analysis, Investigation, Methodology, Visualization, Writing – review & editing. MK: Conceptualization, Data curation, Formal analysis, Funding acquisition, Investigation, Methodology, Project administration, Supervision, Visualization, Writing – original draft, Writing – review & editing. DE: Conceptualization, Data curation, Formal analysis, Funding acquisition, Investigation, Methodology, Project administration, Supervision, Visualization, Writing – original draft, Writing – review & editing.

## Funding

This work was supported by NIH NIDCD grants R01 DC004848 (to DE), P30 DC010362 (to Steven Green) and R01 DC002780 (to MK), and NSF grant 2037828 (to Alan Kay, DE and Zahra Aminzare).

## Conflict of interest

The authors declare that the research was conducted in the absence of any commercial or financial relationships that could be construed as a potential conflict of interest.

## Publisher’s note

All claims expressed in this article are solely those of the authors and do not necessarily represent those of their affiliated organizations, or those of the publisher, the editors and the reviewers. Any product that may be evaluated in this article, or claim that may be made by its manufacturer, is not guaranteed or endorsed by the publisher.

## Abbreviations

IFT, intraflagellar transport
JO, Johnston’s organ
MRP, mechanoreceptor potential
SEP, sound-evoked potential
TEP, transepithelial potential
