# Supplementary material for: The retrograde IFT dynein is required for normal function of diverse mechanosensory cilia in Drosophila
Source: Front Mol Neurosci. 2023 Sep 22;16:1263411. doi: 10.3389/fnmol.2023.1263411 (PMC10556659; doi:10.3389/fnmol.2023.1263411)
Supplement: Supplementary file 1 [file Data_Sheet_1.pdf]

Sharma et al. (2023) “The retrograde IFT dynein is required for normal function of diverse mechanosensory cilia in *Drosophila*”. Frontiers in Molecular Neuroscience.

SUPPLEMENTARY MATERIAL

Genetic analysis of *btv* alleles

1. *btv¹*

As described in the main text, we isolated *btv¹* (originally called *btv⁵ᴾ¹*) in a mutagenesis screen (EberlDuyk and Perrimon, 1997) for mutants with a reduced response to courtship song. This mutant was induced with ethyl methanesulfonate (EMS), which usually generates single nucleotide mutations, with small deletions induced an order of magnitude less frequently.

The *btv¹* mutation was mapped to the 36E1-3 polytene chromosome region based on failure to complement two deficiency chromosomes, *Df(2L)TW119* and *Df(2L)TW201* (EberlHardy and Kernan, 2000), making *N-cadherin* (*CadN*) the left limit and *reduced ocelli* (*rdo*) the right limit (Fig. 5, Table 1) based on breakpoints and complementation. To refine the *btv* map position, we used *P*-induced male recombination (PIMR) (Chen et al., 1998) of *btv¹* relative to a *P*-element insertion in the vicinity, *KG08320* (Fig. 5B). Twelve recombinants for flanking markers *Sp* and *pr* were recovered (Table S1). The *btv¹* lesion maps unequivocally to the right of *KG08320*, as all flanking marker exchanges aligned perfectly with the btv phenotype. One *Sp⁺ pr* recombinant, designated *P2*, had Minute bristles, suggesting a large deletion to the nearby *M(2)36F* locus to the right. Complementation tests confirmed that *btv*, the male sterile locus, *rdo* and *M(2)36F* are all deleted in *P2*, again consistent with *btv* mapping to the right of *KG08320*. This provides a left boundary for *btv* and rules out the *CadN2* candidate (Fig. 5B). Two *P*-element inserts (*KG03741* and *KG05889*, not shown) into the *rdo* gene (CaldwellFineberg and Eberl, 2007) provide a right boundary because *Df(2L)B11* (Fig. 5) is btv⁺ but rdo⁻, while *Df(2L)M18* is btv⁻ and rdo⁺, indicating that *btv* is entirely to the left of the *rdo* locus. Thus, among annotated genes, the candidates for *btv* are limited to *Dync2h1* and its anti-parallel overlapping gene *CG5674*, as well as four small predicted genes between *Dync2h1* and *rdo* (Fig. 5B,C).

2. Transposon insertions: *btv^k07109*, *btv^BG01771*, *btv^f06878*, *btv^f06884*

Several transposon insertions became available through insertional mutagenesis projects. A *P*-element chromosome, *k07109,* (Spradling et al., 1999, Török et al., 1993), contains two *P{lacW}* insertions, one at polytene position 25F and another at 36E, as determined by polytene chromosome *in situ* hybridization (Spradling et al., 1999). This chromosome failed to complement the hearing defect in *btv¹* and all deficiencies in Fig. 5A that uncover *btv¹*. We recovered a recombinant, *k07109b*, that removed the 25F *P{lacW}* insertion, but retained the *btv* lesion. This allele is also called *btv²* (Table 1; Caldwell et al., 2003). This chromosome was white-eyed, suggesting that the remaining *P{lacW}* insertion in 36E may be incomplete, consistent with another report (Mancebo et al., 2001). Nevertheless, plasmid rescue products recovered with flanking genomic DNA confirmed that the plasmid sequences in *k07109b* were still intact; this is consistent with the reported chromosome *in situ* hybridization results with plasmid sequences as probe. The flanking sequences (not shown), though consistent with a 36E location, corresponded to the *fasciclin III* gene, located several genes proximal to the *btv* region (but also not in the nearby *bicoid stability factor* (*bsf*) gene, which is also affected in this chromosome (Mancebo et al., 2001)). Thus, the *btv^k07109* lesion potentially arose as a “hit-and-run” mutation during the dysgenic mobilization event, without leaving a molecular tag in the *btv* gene. We identified the lesion in the *btv* gene through Mismatch Endonuclease Arrays (MENA) (Comeron et al., 2016), as a single nucleotide deletion in exon 22 resulting in a frameshift that introduces an early stop codon (Comeron et al., 2016). This position is in the middle of a large intron in the overlapping gene *CG5674* on the opposite strand, and therefore unlikely to affect its function (Fig. 5C).

Another *P*-element, *BG01771*, is inserted into the *Dync2h1* intron separating exons 13 and 14 (Fig. 5C). This *P*-element construct contains a promoter-less Gal4 sequence with a splice acceptor, along

with a polyA-minus mini-*white* sequence with a splice donor (Lukacsovich et al., 2001). If the *P*-element inserts into a gene intron, the Gal4 would be under the control of the endogenous promoter, resulting in Gal4 expression wherever the gene is expressed. At the same time, the mini-*white* gene would adopt that gene's polyadenylation sequence, allowing expression of the mini-white marker. The expressed eye pigmentation confirmed the presence of the *P*-element in the *BG01771* strain, and PCR analysis verified its insertion site. The insertion site, which lies in the predicted 3' UTR of *CG5674* (1,035 bp downstream of the predicted stop codon and only 260 bases from the transcript end), is unlikely to disrupt *CG5674* unless transcript stability is affected. Conversely, relative to *Dync2h1*, the *BG01771* insertion site is within an intron near the middle of the coding region of the gene. Introduction of a splice acceptor, stop codons and an internal ribosome entry site for the Gal4 sequences is expected to truncate the Dync2h1 protein and, if it encodes *btv*, should produce the btv phenotype. However, despite the fact that, as we show in this paper, *Dync2h1* does indeed encode the *btv* gene, the *BG01771* insertion produces no detectable btv phenotype, as assayed by auditory electrophysiology (Table 1), and by the Drop Zone Assay (DZA) (Table 1; Fig. S2). Furthermore, *BG01771* complements btv^1 and btv^2 in both of these assays. Finally, Gal4 expression, which should be under the control of *Dync2h1*, fails to drive any significant expression of UAS-lacZ or UAS-GFP (not shown).

To determine whether the btv^1 lesion maps left or right of the *BG01771* insertion, we again conducted PIMR. Of nine recombinants recovered (Table S1), seven are consistent with *btv* mapping to the right of *BG01771*. However, two exceptional recombinants, one Sp^- btv^- pr^+ and one Sp^+ btv^+ *pr*, were recovered (Table S1). A lesion that inactivates the *btv* gene may explain the former, but the latter is more difficult to explain this way. More likely, these two recombinants represent gene conversions, which could only occur if the btv^1 mutation is within a gene-conversion-tract distance from the *P*-element. PIMR initiates by a double-strand break at the *P*-element end, and is resolved by strand-recision, strand-invasion and repair. During this process, sequence differences, including substitutions, insertions and deletions, are efficiently converted if within the gene conversion tract length of less than 2 kb (Gloor et al., 1991, Preston and Engel, 1996). Therefore the btv^1 mutation may be very close to the *BG01771* insertion site, likely to the right (based on the majority, 7 of 9, PIMR recombinants).

In the piggyBac insertional screen (Thibault et al., 2004), *f06878* and *f06884*, two insertions of the WH element in the *Dync2h1* and *CG5674* region were recovered. One, *f06878*, is inserted just 6 bp left of the *BG01771* insertion site (Fig. 5C, Table 1). Like *BG01771*, *f06878* is within the *Dync2h1* intron between exons 13 and 14, and in the 3' UTR of *CG5674*. Also like *BG01771*, *f06878* has no detectable btv phenotype. In contrast, the *f06884* insertion fails to complement btv^1 and btv^{07109b}. It is inserted in exon 23 of *Dync2h1*, which is also in the first intron of *CG5674* (Fig. 5C; Table 1). That this insertion is responsible for the btv mutant phenotype was tested by making piggyBac transposase-mediated excisions. Three such excisions all reverted the btv mutant phenotype to the wild type (data not shown).

Together, these insertion alleles point to either *Dync2h1* or *CG5674* as the gene responsible for *btv*, and provide evidence against the four small genes to the right. However, because *Dync2h1* and *CG5674* are strongly mutually nested (Fig. 5C), it is imperative to distinguish them. Therefore, we took three approaches to distinguish these genes: generating imprecise excisions of transposon inserts, generating precise FLP-FRT mediated deletions, and sequencing the btv^1 allele.

3. Imprecise excision: $btv^{1.f234}$ derivative of *BG01771*

Screening for *P*-transposase-mediated excisions of the *BG01771* insertion resulted in many w^- derivatives. Unfortunately, none of these deleted any *Dync2h1* coding sequence; the majority of recovered events were partial excisions of the P-element, while the others appeared to be precise excisions. Because of the propensity for chromosomal rearrangements to occur by recombination between *P*-elements during hybrid dysgenesis, we generated heterozygotes between *BG01771* and the nearby *KG02815* insertion, in the presence of *P*-transposase. This approach, intended to generate a precise deletion between the insertion sites of *BG01771* and *KG02815* (Fig. 5B,C), instead yielded a cluster of three (later found to be identical) deletions. No flanking markers were used, so we cannot verify the mechanism of deletion generation, but the most likely event is a simple imprecise excision of the

BG01771 element without flanking exchange. The deletions, represented by *btv*$^{1.f234}$, (which we here rename *btv*^{4}) remove 3196 bp and insert a G nucleotide; they extend from 1377 bp left of the *BG01771* insertion site rightward into the intron between exons 11-12 of *Dync2h1* (Fig. 5C, Fig. S3). Thus *btv*^{4} deletes two exons from *Dync2h1* and introduces a frameshift. The *btv*^{4} lesion is predicted to remove 60 C-terminal amino acids from the *CG5674* gene product, replacing them through frame-shifting with 24 unrelated amino acids before termination. The three lines represented by *btv*^{4} exhibited sedentary locomotor behavior, elevated DZA scores and electrophysiological deafness similar to the other *btv* alleles (Table 1). This further supports *Dync2h1* or *CG5674* as *btv*, but does not distinguish between them.

4. FLP-FRT-mediated deletions

With the availability of FRT-containing piggyBac insertions in the region, we used FLP-mediated recombination between nearby piggyBac insertions to generate precise deletions (Parks et al., 2004). Deletion #1 (del#1; Fig. 5C) was generated between the *f06884* insertion near the 3' end of *Dync2h1* and the *f06319* insertion to the left of it. This removes about 104 kb including the first predicted exon of *CG5674* and much of the last three predicted coding exons of *Dync2h1*. Deletion #2 (del#2; Fig. 1) removed about 19 kb, the region between *f06878* rightward to *f06603*, which includes the first 13 exons of *Dync2h1*, as well as part of the 3' UTR of *CG5674*. Both del#1 and del#2 result in deafness and fail to complement other *btv* alleles. These results eliminate the four small genes to the right as candidates, leaving only *CG5674* and, more likely, *Dync2h1*.

5. *btv*^{1} deletes part of *Dync2h1* exon 13

Because *btv*^{1} was induced with EMS (EberlDuyk and Perrimon, 1997), we expected to find a single nucleotide mutation. Thus we tried to PCR-amplify DNA around the *BG01771* insertion site from *btv*^{1} homozygotes for sequencing. However, several primer pairs which amplified products from the 40AG13 background strain failed to produce amplicons from *btv*^{1} DNA, suggesting a possible deletion around exon 13. Southern blot analysis using 3 different restriction enzymes (data not shown) was consistent with a ~400bp deletion. PCR using primers Se13F (in exon 13) and Dhc9R (in exon 9) produced a PCR product ~400bp smaller in *btv*^{1} than in *40AG13* DNA (Fig. 6A,B). Sequencing this product revealed a 401bp deletion along with a 6bp insertion (Fig. 6C). The deletion removes 125bp of *Dync2h1* exon 13 and part of the intron between exons 12 and 13. Because this deleted region excludes any *CG5674* sequences, the *btv*^{1} lesion firmly establishes *Dync2h1* as *btv*.

Supplementary Figures

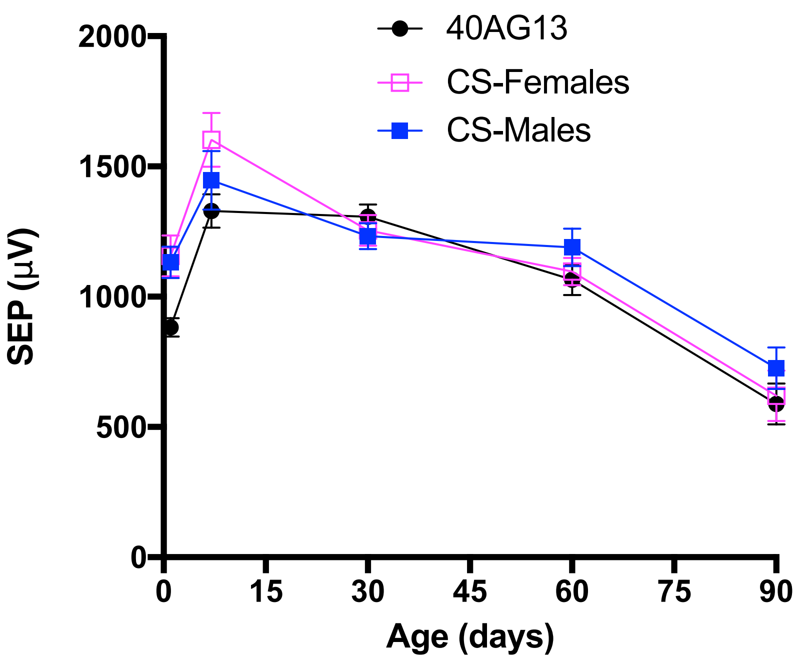

Figure S1. Age-dependent hearing loss in *Drosophila*. SEP recordings from the antennal nerve of Canton-S wild-type flies and the 40AG13 control strain. Depicted are the means ± SD. For days 0 to 60, each data point represents 24-45 antennae, while at 90 days, each point represents 14-20 antennae.

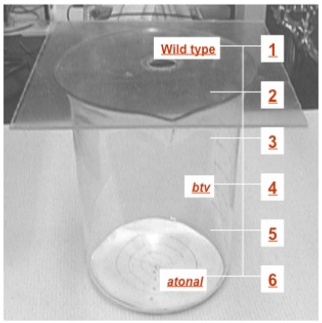

Figure S2. Drop Zone Assay. The Drop Zone Assay (DZA) is a behavioral assay to quantitatively assess motor coordination that includes flying and walking behavior. Flies are introduced individually or 5 or 10 at a time into a 4-liter beaker, through a 1 inch hole in a plexiglass cover. After observing for 1 minute, flies are given a score between 1 and 6 as follows: 1 = fly flies and lands on the plexiglass cover, 2 = fly flies, lands on the side of the beaker and walks up to the cover, 3 = fly walks more than halfway up the wall of the beaker, 4 = fly walks less than halfway up the wall of the beaker, 5 = fly lands on bottom, walks around but does not walk up the wall of the beaker, 6 = fly lands on bottom and stays in position. Scores are averaged, and typical scores for wild-type flies are between 1 and 2, *btv* mutants typically score around 4, while *atonal* mutants score close to 6.

A

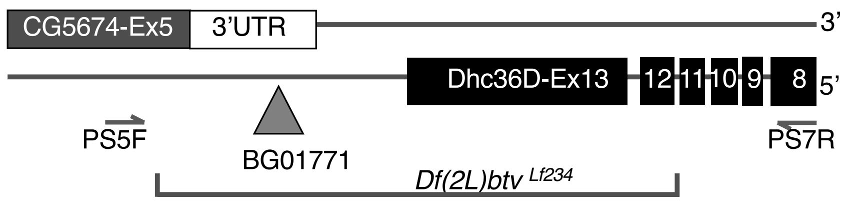

B

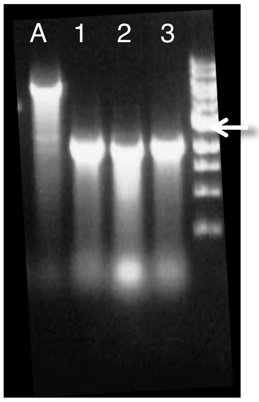

C

CCCGCCCAGCCACCTCCGCAGCAACAGCATCT
TTTCAGCGCCCATCAGCACCAGCAGCAGCAAC
AGCAGCAGCAGCAGCAGCAACATCATCTGCAG
CAGCA/ACAGCACTGCAACATGTTCCAGCATA
TGGCC…

~3 kb deletion

…GGGGAATTACCATGCACTCGGCAAAA/ATGT
GGGTGTACATTTTTCTTTTGGTAAAAAACTTA
CATTGCGGTAAGTTTTTTTTTTCAATAT

Figure S3. The *btv*^{Lf234} deletion removes 3 kb affecting both *Dync2h1* and *CG5674*. A) Map of central exons of *CG15148* (*Dync2h1*), with symbols as in Fig. 6. Positions of primers PS5F and PS7R used to confirm the deletion are indicated. The approximate position of the *btv*^{Lf234} deletion is shown by the bracketed region. B) Three deletion lines that arose as a cluster from a single dysgenic fly show the same deletion of about 3 kb. Panel shows PCR amplicons using primers shown in A to generate about a 5 kb band in the 40AG13 control (lane A), but only about 2 kb in the *Lf234*, *Lf245* and *Lf249* lines (Lanes 2, 3 and 4). C) Sequence analysis of the Lf234, Lf245 and Lf249 lines revealed identical deletions with 3196 nucleotides deleted and replaced with a single G nucleotide. Only the sequences of the ends of the deleted segment are shown, and the deletion breakpoints are indicated by forward slashes. The sequence corresponding to the PS5F primer is underlined.

MTQRTKFIINTSVEYFSARLPGSELDEQTAMILEFLEQEELTVISAVHSRSDGRIRFHHRIPNEELCLLFYKVPQVGHHR
KEDGSEPLLGILTLEGGMVKSIYNSVSRVFSPSANSARRSEYGPELSGILENLHQNLGSSLGLPQSGITSLRNEIKYWQQ
KLGQKSSSRLDREAAQVFIGVLENMEKQISTIDGANPSGTIEEFLDHAHTNLDELWRLPYNYPQQRMADLLDIIGKRLLE
VCLTQLLAEDVWSLNSSHVNNLMSQSIDTVDAWIQLCDSLTRLFWPNYVKHPWLGDSHVPKRGQQFKERLSEIRSIKQLY
KQIATLLEDTELQEMFQEQAPFTEFNIFDTSSLGQNKWHKALQHFEQVLQPIDERIAAALKRQLHNHLSNPRQVIFIFSK
YETLIQRPAVLELLTIEREQFLQSLHILLQDLRKAMVDSNMEPDTGHLSVICNECRWLKVVQHQIQEIEKVSHLISGREG
FDKINKAVQEIKEETESLLRTNFEIWSGQCSTAVKSGELRLRDDQAVVKFEKEGRQLMRVTFNPKLVTFCQDVREFENLG
YNVPLELRAAATHAAKYMCYARRLQQIATFHNTIGDRMIPCQRPIMLKNALELQRLVQSETVAWQDESSVQRYVDILQAA
VSKLSSDNTLLVGYHEQAKRSVLKLMSTDLFTQNQIWKDELRHLRELVATLERQGYTHLDAFKLHWDHQLYKVLEYQYIL
GLLDMNNKLPDIHVKLVLRQRELVFSPPEEEIRELYFSQLRRFIERPCNFHGLSEHSQELFKSMVTVNRHHFGPLYQRAA
ELFDKLQDFKTIWLPWIALGCVDVDQLCGIHLNEAGDWDRNFRSCKHFSQQLAKLQQAEESIDCIVINVLPLRSDIEYIS
RRYWESLANSLRTAILTDVSLIQEFLQSALQFLQNVPMDEGSISQSGMKYEKIMSKLPQIEKTLEAVRAKDSCLGGWCKE
RVTALSGILLQWEQLQPLLENHAVILQRQVDMIKNQAETQLQNLKNEAEKFLLRWESTISELEANEHSTLDVFKERRLHW
QQLQEKKTQLLEECSKFNMEFPAEMLTPFTEIEEQMEKQSKQWQVYDSFLTELQPVLHEEWAIYRRRPYVLNEFIGKWEG
SVHASIDLPSKRIRQQVEQLQSALPILQQLQSESLSERHWARIFQLLNHKETKPLHSILLQDILQDFDVLQSAAQEISSI
VRQASSEQIVRQALIELDQWSVTAQLKLITRTDASGQSVSLIKDYQEVLNKIGDNQSLLQSANNSAAFESFSDQAELWES
RLNTLDALLSSLNHSQRRWVYLEPVFGSGTLQHEQALFKRIDKDFRFVMREIEMDPRVTSLTKINNITTIVNALETQLAR
CQQNLMSYITDKRNSFPRFYFLGDDDLLELLGQASKDAEVIQRHIRKLFPGCHSLSIRQVGPNPATSSDVNQYSITSVHS
AEGDELKLSQPVEMKGDIERWLNQLVTVVQDTLRDQIYECYTGTTGGSDNLDEKILKKYASQVLATARALHFTRQAEQAI
GSMSLGKLKQQLKDEITHLAALKNKSENGTLISLKLRALLLDLVHYSGVTEQLQKHNVVHTSDWHWLCQLRYYLGKKGGT
SGEVNANRQVCVRMVYAEFEYAYEFLGQASKLVHTRLTHRCYLILTQAMHMGLGGNPF**GPAGTGKT**ECVKALGAMLGRLV
LVFNCDENVDTESMSLILTGLARCGAWGCFDEFNRLQEATLSSISMLIQPIQSALKERANSVQIGERQIQLNQHCGIFVT
LNPAGAEYGGRQKLPGNIQALFRPIVMQQPEPGEIARVMLFVEGFTEASAIASRIVELFELSGKMLSAQRHYDWGLRELK
TVLMVCGEGLRDQLTSEDNNQSSANFEMSVVVRCLRSSTMSKLAPHDVNRFEMLLRNVFPEIGSSPAPETQLHQSLSAAF
AQLGLCRSERQIEKALQLHEQLQKRMGVVLV**GPPGCGKS**TIISLLKQALCGTQLKVHTISPKSMSRIQLLGRLDADTRQW
QDGVLTHTAVAVNQESSQVHSWIVCDGSIDPEWIEALNSVLDDNKLLTLPSGWRIQFGSNVNFIFETDDVRHASPATISR
MGIVNMSYDYYPADGILKHELSKEPYGDLLQSYVDGKFQYAVNWIESQFLLTNHLPGINRAHLLRSLLLQLHGTQSLEEY
GAATLRALFGYMPNDRQREFAQLILKHANLYVANPNYAELTHYESSRNSLEQYAVDAIETPEKGSQLIITSYMKSYLDIL
ETLLKTQGTRLPPFMLI**GPSGSGKT**LLLQRAVLENSGYQLATINCSTQLTPRYILHTLKTHCVTVSGIKGREYRPKQARL
VLFMKNLDLCQQDSWGACEVVELLFQLAQRGGFYAENLEWIGVSGLQLCASIGGNTGKIAPRYFAINQFVRVSRPTSQDM
LEIVQRRLEPLLEEHFRGSENRGRSGVNLQHVSESLMDCFEKLQATFTNVGGRQAHYQFSPKCIMKLLDALVFYPASDFN
EALYCELLGMFRDRLISEEHVQQFEGILKQTMRKYYGKEKVFFVPKSPKSRGHLHCLTHDEWMEEVQRQVTICNTENYSI
TAPITEELLSHVARITRVLSRTDAHMLIL**GQSGGRHL**DAIFTAATFQEAKVVTLQGGPSYDLTDFYNDLKVAMQTAALEQ
QMSYLLIEQCWLSYVPDILKPIEALLEGSEILELFGDDLETVASTLKQAAQLEGYQESMGTYFLKRARDYLHIIIVLDPN
SAKVQDYFNNFPALHRQMDLLYVRGESRETIAILPKQFIELLNESIAGGGSGRGKVPTCSHFADISDELPSEETSQRYYQ
LIRTYFHMYNNAANEIDQRLGKLQMGVDKLASAHALVDTLKSNAAAQEQALGEKRQLANEALEMISFTMRNANEQKSSML
ELKQQTQKSSEQLKIRQKEIQQELAEVEPILAEASNAVGQIKSEALSEIRSLRAPPEAVRDILEGVLRLMGIRDTSWNSM
KTFLAKRGVKEDIRSLDPARISPENCEAVERLLLAKGDSYEAKNAKRASAAAAPLAAWVQASVRYSRVIQSIKPLEREQN
ELQKNLNAAEDEMQELASGLDDVDKRVKQLSAKLQTYTQEAAVLELKLQEASGTLQAAELLVEKLSAEYTTWSLQLTELK
KAHKTLDAKTLLIAIAINYCAGLGLEQRCSSLKRLAADFHLPSDFDLRGSLLTEQQQIIWESQGLARDAQIIESAALLRE
MLSLPYGACPIPLLLDPTQTAAAWLMAHLKGSGRPCELTTHGNDRLPYQLELAVRFGKTLLVTDCEQLRPPVLQLLQGHV
FVRFNKRQLAIGSKLVDLHESFQLVLISKSHRLDLPEEQRSQLNVLKFTVTAAGLADQLMSKAIVLKNGELEQQRIELLQ
KEGHLLKQRMEMQDKLLEQLSKSEGDILKNEQLLESLNEIKQGSTQIDEALKQSGQIRDTLLAQFGSLRELSSRAATFYA
GLIQGYELSPLVYIELFLGALSKSQRDESKVYDCLVRSVYMNLARATSRDSQLSLSLWVCHQAYPDRLNPKEWELFVNNF
MGSSDGSMVLSQLGKLPDCMPKEAQLKLAMLLQLFPDLRSKLQLEKDYIWRGFIEAQADDVLPALGSSFQRVLIAQIFRP
DLMLHQLRKVSSDLLGISPDASTQPSVEQLLQQSSCDRPILMVSHAENDPTTELRKWANQKYREMAIGKGVEKRVLSEMR
QAAIDGHWLCVKNVHLVPEFLTQMERELSEIQKSKDFRLWLLCESTEGFSEAAIYKCLKVRYEQPKGLKQIVMRLLQNFA
AEQDQKLKNQPKSLKMRLVYFVLTAVLQQRRQFIPQGWSKYYEFGEADLKAALGILRMMDQQLNSGKCDWLLMQRLSEAL
AYGGRVNNQRDLEILTTYLNQFCSADVLSNRWSPLGLSLSIPTSGQLQDYYAALEKLPDTDEPSMYGLANQAQQQREIEQ
AKRVIKELRGLHYGRGLAKDSGGAGESKGNDQLTGRQKLEQQIKPLLNLWRKLAASCTIIQTMKEAKTDVGESPWALFVL
AELKLGADLYGIVHQTLSQMHAWLKESQEVDGSTLRTLAEQQIPASWLKLWPGPGSNSAVDFLRALIVRAQAAELRFREQ
MHLDFVEDINFVQVFNCENLLSCLKLLQSRKLAVSTERLELQTCGSSNLESDSSDIILKLAPLK//

1. VSKN (if no exon 25)

2. IDGAQSGMGKSNPFYIKYKIKDEVQNVSHTTTSSSKYGKNSLYSTQNAESKPKLPLYSRSSRDKLICHLNVDIVTGT
AEQILLAGTALIVEDY (with exon 25)

Figure S4. The amino acid sequence of Dync2h1 encoded by the *btv* gene. Two possible translation start sites (see Fig. 5) are indicated, with the first generating a longer amino terminal end (red typeface). The selection of start sites has not been confirmed experimentally. The four predicted P-loop motifs are in bold underlined letters. At the carboxyl terminal, two possible terminal peptides are shown, depending on whether predicted exon 25 (see Fig. 5) is actually used. For the predicted form including the longer N and C termini, the predicted size of the entire protein is 481.45 kDa, consisting of 4237 amino acid residues.

```
                          P-loop 1
Hsap  DKCYLTLTQAMKMGLGGNPYGPAGTGKTESVKALGGLLGRQVLVFNCD 1716
Rnor  DKCYLTLTQAMKMGLGGNPYGPAGTGKTESVKALGGLLGRQVLVFNCD 1716
Tgra  DKCYLTLTQGMHMGMGGNPYGPAGTGKTESVKALGGLFGRQVLVFNCD 1724
Cele  DKCYLTLTQAMYMGLGGNPYGPAGTGKTESVKALAALMGRQVLVFNCD 1664
Crei  DKCYLTLTQGMALGYGGNPYGPAGTGKTESVKALGQALARQVLVFNCD 1770
Agam  HNCYLILTQAMQLGLGGNPFGPAGTGKTECVKSLGAMLGRLVLVFNCD 1625
Dmel  HRCYLILTQAMHMGLGGNPFGPAGTGKTECVKALGAMLGRLVLVFNCD 1686
      ..*** ***.* :* ****:*********.**:*.  :.* *******


                          P-loop 2
Hsap  IKKALELYEQLCQRMGVVIVGPSGAGKSTLWRMLRAALCKTGKVVKQY 2006
Rnor  MKKALELYEQLRQRTGVVIVGPSGAGKSTLWRMLRAALCKIGKVVKQY 2006
Tgra  VKKALELYEQLRQRMGVVVVGPSGSGKSTTWQILRAALNNTGQVVKQY 2016
Cele  MEKVFQLYEQMRQRIGVVVVGAAGSGKSTIWKILQRSLILTKKPLKVT 1948
Crei  IDRMLQLHLACEQRIGVIIVGPSGSGKSTLWELLEKAYERLGRKPIVY 2061
Agam  VEKCLELQAQLQKRMGVVVIGPPQSGKTTIIALLKEALIAQGQIIRIH 1914
Dmel  IEKALQLHEQLQKRMGVVLVGPPGCGKSTIISLLKQALCGT--QLKVH 1977
      :.: ::*     :* **:::*.. .**:*   :*. :


                          P-loop 3
Hsap  GLDYFKPWLSSDTKQPFILVGPEGCGKGMLLRYAFSQLRSTQIATVHC 2318
Rnor  GLDYFKPWLSSETKQPFILVGPEGCGKGMLLRYAFSQLRSTEIATIHC 2318
Tgra  CLDFFNPWLEADNRQPFILVGPDGCGKGMVLRHCFAQLRSTQIATIHC 2328
Cele  YSDIIGSWLQSGNRESFLITGTTGCGKQQLLKHCFQNDPESQLASLYC 2253
Crei  NLLMMAPWFK--NRDPFLVVGPEGCGKGALLDYCFKRIMGVQVAVVNC 2361
Agam  DLLKHIIASK-E-RYVALLVGPSGNGKSLLLQTIVSEFSGYQLVTINC 2139
Dmel  DILETLLKTQGTRLPPFMLIGPSGSGKTLLLQRAVLENSGYQLATINC 2285
               .       :: *. * **  :*   . .    ::. : *


                          P-loop 4
Hsap  EYMSRIDRVLSFPGGSLLLAGRSGVGRRTITSLVSHMHGAVLFSPKIS 2682
Rnor  EYMSRIDRVLSFPGGSLLLAGRSGVGRRTVTSLVSHMHGAVLFSPKIS 2682
Tgra  DHVARVDRVLTQPRGSLLLAGRSGVGRRTAASLVAHCHRTELFSPNLS 2688
Cele  FFCACIDRVLTGPGGHLFLPGRPGFGRRDSVRLVAHMHNIQVFSPPVT 2592
Crei  ERVSRFDRVLSQQGGSLLLCGNSGVGRRSLMLLLAYMHNMDFITPKMT 2706
Agam  ETIASIARALSRPYANLVMIGRAGSGRLQALYTACTMLNVRVAFPQMS 2467
Dmel  SHVARITRVLSRTDAHMLILGQSGGRHLDAIFTAATFQEAKVVTLQGG 2617
         : . *.*:   . :.: *..*  :       .      .
```

Figure S5. Conservation of four P-loops in Dync2h1. ClustalW was used to compare several Dync2h1 isoforms. The P-loop motifs are underlined and their positions are shown within the amino acid sequence context. The numbers at the end of each line reflect the amino acid position of the last residue in the line relative to the full-length annotation. The gi (gene identifier) numbers for these sequences are mentioned in the text. Only the sequences for the highly conserved P-loops are shown. Hsap, *Homo sapiens* gi:311033479; Rnor, *Rattus norvegicus* gi:12711694; Tgra, *Tripneustes gratilla* gi:17019507; Crei, *Chlamydomonas reinhardtii* gi:75337416; Cele, *Caenorhabditis elegans* gi:74963878; Agam, *Anopheles gambiae* gi:158298344. Small and hydrophobic residues (AVFPMILW) are shown in red, acidic residues (DE) are shown in blue, basic residues (RK) are shown in magenta, and residues with hydroxyl, sulfhydryl or amine side chains (STYHCNQ), as well as glycine (G) are shown in green.

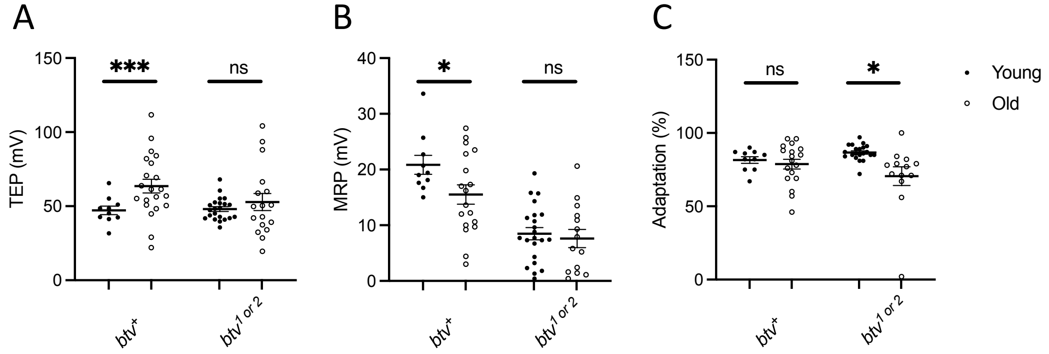

Figure S6. *btv* mutants do not undergo accelerated age-dependent degeneration in bristle organ function. A) TEPs in control flies are significantly enhanced in old flies (30 or more days old, open circles) compared to young flies (less than 5 days old, solid circles), but not in *btv* mutants. B) MRPs in control flies are significantly reduced with age, but age does not further reduce MRPs in *btv* mutants. C) Adaptation is not affected by age in control flies but shows a slight though significant decrease in *btv* mutants. For A-C, data from *btv*^{1} and *btv*^{2} are not significantly different and are thus pooled.

Supplementary Tables

Table S1. P-induced male recombination

Parental genotype	Progeny screened	*Sp pr*$^+$ recombinants	*Sp*$^+$ *pr* recombinants	Interpretation
Sp btv^1 *pr rl cn*/ *Sp*$^+$ *KG08320 pr*$^+$	~2500	8 all btv$^+$	4* all btv$^-$	*btv*^{1} lesion is to the right of *KG08320*.
Sp btv^1 *pr rl cn*/ *Sp*$^+$ *BG01771 pr*$^+$	5966	5 4 btv$^+$, 1 btv$^-$	4 1 btv$^+$, 3 btv$^-$	*btv*^{1} likely to the right of, but very close to, *BG01771*.

* One *Sp*$^+$ *pr* recombinant is a large deletion from the KG08320 insertion site extending rightward to at least *M(2)36F*, including *btv* and *rdo*. We named this *Df(2L)P2*.

Table S2. Polymorphisms in the *btv* gene coding sequence

Annotated Feature	40AG13	w^{1118}
R237		R237R
A409	A409T	A409T
F503	F503F	F503F
Q602		Q602R
Q614	Q614Q	Q614Q
S619	S619S	S619S
L636	L636L	L636L
L729	L729L	L729L
N769		N769N
Q807	Q807E	Q807E
D823	D823D	D823D
K936	K936E	
G955	G955A	G955A
Q1000	Q1000Q	Q1000Q
L1013	L1013L	L1013L
H1027	H1027H	H1027H
L1038	L1038F	L1038F
S1080	S1080S	S1080S
D1087		D1087G
V1096	V1096I	V1096I
E1153	E1153D	E1153D
S1154	S1154S	S1154S
E1196		E1196G
Q1208	Q1208H	Q1208H
T1232		T1232A
I1242	I1242I	I1242I
N1263	N1263K	N1263K
R1330	R1330R	R1330R
K1670	K1670K	K1670K
T1806	T1806T	T1806T
N2127	N2127K	N2127K
L2139	L2139F	L2139F
N2205	N2205N	N2205N
I2238	I2238L	
F2456	F2456S	
E2706	E2706E	
H2744	H2744H	
Q2805	Q2805Q	
F3564	F3564C	
R3568	R3568Q	
S3569	S3569I	
N3702	N3702N	

Note: Polymorphisms found in the two lab strains *40AG13* and w^{1118} are listed according to their effect on the *Dync2h1* amino acid sequence, encoded by the *btv* gene, with respect to the annotated sequence generated in the fly genome project. Synonymous mutations are listed in black typeface, non-synonymous changes are shown in red.

References cited in Supplementary Material

CALDWELL, J. C., FINEBERG, S. K. & EBERL, D. F. 2007. *reduced ocelli* encodes the leucine rich repeat protein *Pray For Elves* in *Drosophila melanogaster*. *Fly,* 1**,** 146-152.

CALDWELL, J. C., MILLER, M. M., WING, S., SOLL, D. R. & EBERL, D. F. 2003. Dynamic analysis of larval locomotion in *Drosophila* chordotonal organ mutants. *Proceedings of the National Academy of Sciences (U.S.A.),* 100**,** 16053-16058.

CHEN, B., CHU, T., HARMS, E., GERGEN, J. P. & STRICKLAND, S. 1998. Mapping of Drosophila mutations using site-specific male recombination. *Genetics,* 149**,** 157-163.

COMERON, J. M., REED, J., CHRISTIE, M., JACOBS, J. S., DIERDORFF, J., EBERL, D. F. & MANAK, J. R. 2016. A Mismatch EndoNuclease Array-based Methodology (MENA) for identifying known SNPs or novel point mutations. *Microarrays (Basel),* 5**,** E7.

EBERL, D. F., DUYK, G. M. & PERRIMON, N. 1997. A genetic screen for mutations that disrupt an auditory response in *Drosophila melanogaster*. *Proceedings of the National Academy of Sciences (U.S.A.),* 94**,** 14837-14842.

EBERL, D. F., HARDY, R. W. & KERNAN, M. 2000. Genetically similar transduction mechanisms for touch and hearing in *Drosophila*. *Journal of Neuroscience,* 20**,** 5981-5988.

GLOOR, G. B., NASSIF, N. A., JOHNSON-SCHLITZ, D. M., PRESTON, C. R. & ENGELS, W. R. 1991. Targeted gene replacement in *Drosophila* via P element-induced gap repair. *Science,* 253**,** 1110-1117.

LUKACSOVICH, T., ASZTALOS, Z., AWANO, W., BABA, K., KONDO, S., NIWA, S. & YAMAMOTO, D. 2001. Dual-tagging gene trap of novel genes in *Drosophila melanogaster*. *Genetics,* 157**,** 727-742.

MANCEBO, R., ZHOU, X., SHILLINGLAW, W., HENZEL, W. & MACDONALD, P. M. 2001. BSF binds specifically to the *bicoid* mRNA 3' untranslated region and contributes to stabilization of *bicoid* mRNA. *Molecular and Cellular Biology,* 21**,** 3462-3471.

PARKS, A. L., COOK, K. R., BELVIN, M., DOMPE, N. A., FAWCETT, R., HUPPERT, K., TAN, L. R., WINTER, C. G., BOGART, K. P., DEAL, J. E., DEAL-HERR, M. E., GRANT, D., MARCINKO, M., MIYAZAKI, W. Y., ROBERTSON, S., SHAW, K. J., TABIOS, M., VYSOTSKAIA, V., ZHAO, L., ANDRADE, R. S., EDGAR, K. A., HOWIE, E., KILLPACK, K., MILASH, B., NORTON, A., THAO, D., WHITTAKER, K., WINNER, M. A., FRIEDMAN, L., MARGOLIS, J., SINGER, M. A., KOPCZYNSKI, C., CURTIS, D., KAUFMAN, T. C., PLOWMAN, G. D., DUYK, G. & FRANCIS-LANG, H. L. 2004. Systematic generation of high-resolution deletion coverage of the *Drosophila melanogaster* genome. *Nature Genetics,* 36**,** 288-292.

PRESTON, C. R. & ENGEL, W. R. 1996. *P*-element-induced male recombination and gene conversion in Drosophila. *Genetics,* 144**,** 1611-1622.

SPRADLING, A. C., STERN, D., BEATON, A., RHEM, E. J., LAVERTY, T., MOZDEN, N., MISRA, S. & RUBIN, G. M. 1999. The Berkeley Drosophila Genome Project gene disruption project: single *P*-element insertions mutating 25% of vital Drosophila genes. *Genetics,* 153**,** 135-177.

THIBAULT, S. T., SINGER, M. A., MIYAZAKI, W. Y., MILASH, B., DOMPE, N. A., SINGH, C. M., BUCHHOLZ, R., DEMSKY, M., FAWCETT, R., FRANCIS-LANG, H. L., RYNER, L., CHEUNG, L. M., CHONG, A., ERICKSON, C., FISHER, W. W., GREER, K., HARTOUNI, S. R., HOWIE, E., JAKKULA, L., JOO, D., KILLPACK, K., LAUFER, A., MAZZOTTA, J., SMITH, R. D., STEVENS, L. M., STUBER, C., TAN, L. R., VENTURA, R., WOO, A., ZAKRAJSEK, I., ZHAO, L., CHEN, F., SWIMMER, C., KOPCZYNSKI, C., DUYK, G., WINBERG, M. L. & MARGOLIS, J. 2004. A complementary transposon tool kit for *Drosophila melanogaster* using *P* and *piggyBac*. *Nature Genetics,* 36**,** 283-287.

TÖRÖK, T., TICK, G., ALVARADO, M. & KISS, I. 1993. *P-lacW* insertional mutagenesis on the second chromosome of *Drosophila melanogaster*: Isolation of lethals with different overgrowth phenotypes. *Genetics,* 135**,** 71-80.
